# What would happen if twitter sent consequential messages to only a strategically important subset of users? A quantification of the Targeted Messaging Effect (TME)

**DOI:** 10.1371/journal.pone.0284495

**Published:** 2023-07-27

**Authors:** Robert Epstein, Christina Tyagi, Hongyu Wang

**Affiliations:** American Institute for Behavioral Research and Technology, Vista, CA, United States of America; Roma Tre University: Universita degli Studi Roma Tre, ITALY

## Abstract

The internet has made possible a number of powerful new forms of influence, some of which are invisible to users and leave no paper trails, which makes them especially problematic. Some of these effects are also controlled almost exclusively by a small number of multinational tech monopolies, which means that, for all practical purposes, these effects cannot be counteracted. In this paper, we introduce and quantify an effect we call the Targeted Messaging Effect (TME)–the differential impact of sending a consequential message, such as a link to a damning news story about a political candidate, to members of just one demographic group, such as a group of undecided voters. A targeted message of this sort might be difficult to detect, and, if it had a significant impact on recipients, it could undermine the integrity of the free-and-fair election. We quantify TME in a series of four randomized, controlled, counterbalanced, double-blind experiments with a total of 2,133 eligible US voters. Participants were first given basic information about two candidates who ran for prime minister of Australia in 2019 (this, to assure that our participants were “undecided”). Then they were instructed to search a set of informational tweets on a Twitter simulator to determine which candidate was stronger on a given issue; on balance, these tweets favored neither candidate. In some conditions, however, tweets were occasionally interrupted by targeted messages (TMs)–news alerts from Twitter itself–with some alerts saying that one of the candidates had just been charged with a crime or had been nominated for a prestigious award. In TM groups, opinions shifted significantly toward the candidate favored by the TMs, and voting preferences shifted by as much as 87%, with only 2.1% of participants in the TM groups aware that they had been viewing biased content.

## 1. Introduction

Research conducted over the past decade has identified a number of new forms of influence that the internet has made possible. Some of these are among the largest effects ever discovered in the behavioral sciences, and they are of special concern because they can impact people without their awareness, because they often leave no paper trails for authorities to trace, and because they are largely controlled by unregulated monopolies [1–3]. Epstein and Robertson showed, for example, that search results that are biased to favor one candidate could shift the voting preferences of undecided voters by as much as 80% after just a single search experience on a Google-like search engine [1], and this effect has since been replicated partially or in full multiple times [4–11]. They also showed that this effect, called the “search engine manipulation effect” (SEME), can easily be masked so that users are unaware that they are viewing biased search results.

In the present paper, we describe and quantify yet another new form of online influence–the Targeted Messaging Effect (TME)–which has all of the most troubling characteristics of SEME and other recently identified forms of online influence [10–14]: it is a large effect; it can influence people without their awareness; it leaves no paper trail; and it is largely controlled worldwide by three unregulated monopolies–Facebook/Meta, Google, and Twitter.

Before we say more about TME per se, we will attempt to put our research on this topic into a larger context. Research on influence over human decision making has been conducted for over a century in multiple fields: business, psychology, sociology, political science, economics, and so on. In political science, for example, Paul F. Lazarsfeld’s classic studies in the 1940s and 1950s demonstrated the important role that “political predispositions” played in determining how people reacted to various forms of social influence, and, ultimately, in helping to determine how people voted [15–17]. Political scientists have also shown how voters are influenced by a wide range of factors, among them being the positive or negative connotation of a political message and the presence of a political candidate in media–newspaper coverage and television ads, for example [18–20]. Recent investigations show how voters are influenced by social media content, the online presence of a political candidate, and the perceived personability of a political candidate across different platforms [21–24].

Economists and business experts have developed numerous models to try to understand and predict consumer behavior [25,26]; once again, recent efforts have focused on how search engines, social media platforms, YouTube “influencers” and other new forms of influence made possible by the internet and other new technologies are impacting consumer choices [27,28]. Psychologists have been trying to understand decision making in broad terms applicable, perhaps, to all aspects of life, and they have been especially interested in recent decades in identifying extremely subtle forms of influence that are largely invisible to those affected [29–31].

We believe that SEME, TME, the Answer Bot Effect (ABE) [11], the Search Suggestion Effect (SSE) [12], and other new forms of influence that our research group has been studying over the past decade are fundamentally different than most forms of influence researchers have been studying over the years. Most forms of influence are inherently competitive: billboards, social media campaigns, television commercials, and print and online advertisements, for example. Even most of the shady forms of influence one sometimes reads about in headlines or novels are inherently competitive: ballot stuffing, the rigging of voting machines, vote buying, and so on [32,33]. Competitive forms of influence usually have little net effect for the simple reason that both (or all) sides can employ them. One manipulation might overpower the others when one side has more resources, but resources can shift over time.

The internet was envisioned by its founders to be a great leveler, giving every individual equal voice and giving small companies the ability to compete with giants [34,35], but it quickly evolved into an array of “walled gardens” [36,37] dominated by huge monopolies, each of which quickly gaining virtually exclusive control over specific forms of influence. Outside the Republic of China, Google (through its search engine and its property YouTube) controls access to most information, and Meta (through its properties Facebook, Instagram, and WhatsApp) guides the majority of online social interactions. TikTok has also become popular, accruing over 2 billion first-time downloads since its release in 2016, and it has even become a platform for “forming political coalitions” among young users [38–40]. Although far smaller than Google and Facebook, Twitter dominates the influential world of microblogging, especially in the United States [41,42].

The current walled-garden structure of the internet is highly problematic from an influence perspective. It means that if one of the large platforms favors one candidate, party, cause, or company, it can change people’s thinking and behavior on a massive scale without people’s awareness, without leaving a paper trail for authorities to trace, and without anyone having the means to counteract the manipulation. To be specific, if Google’s search algorithm boosted content in search results that favored Candidate A, unless systems were in place to capture such content–all of which is ephemeral–no one would ever know that this bias existed, even though, in a national election, it could conceivably shift the voting preferences of millions of undecided voters [1–3,10–14]. Even more disturbing, no one could counteract such bias. To put this another way, although two opposing campaign groups might battle each other to try to boost their visibility in search results or in YouTube sequences, *no campaign organization has the means to counteract an action taken by or a policy implemented by the platform itself*–by an executive, a rogue employee, an unattended algorithm, or some combination thereof. The problem worsens when these monopolies favor the same candidate or cause; patterns of campaign donations documented by organizations such as OpenSecrets.org in recent years suggest that major tech companies might in fact be politically aligned [43–45].

TME itself was presaged in a widely-read *New Republic* article by Harvard legal scholar Jonathan Zittrain [46]. As he noted, on Election Day in the US in 2010, Facebook sent go-vote reminders to 61 million Facebook users and, based on a nationwide analysis of voting records, subsequently concluded that its go-vote prompt had caused about 340,000 more people to vote than otherwise would have [47]. The prompt successfully nudged 0.57% of Facebook’s sample of eligible voters. That might not sound like much, but that proportion could easily swing a close election. Recall that Donald Trump won the Electoral College vote in 2016 because of a combined vote margin of only 79,646 votes in three US states [48]. If Mark Zuckerberg, CEO of Facebook, had elected to send vote reminders exclusively to supporters of Hillary Clinton on Election Day in 2016, that might have boosted the Clinton vote nationwide by more than 450,000; that number is based on a simple extrapolation from Facebook’s 2010 vote manipulation [49].

Zittrain’s concerns were legitimate, but, for four reasons, we believe that “digital gerrymandering” is an inappropriate label for this type of manipulation. First, gerrymandering–the relatively permanent redrawing of voting districts–and targeted messaging–the sending of consequential messages to only a subset of a larger group–have at best only one superficial characteristic in common: they each divide up a population in a way that serves the needs of an empowered group. But gerrymandering is a visible and relatively permanent manipulation–so visible and heavy handed that it is often challenged in court [50]. TMs sent to a subgroup online, however, are ephemeral. They impact people and then disappear. They are stored nowhere and cannot be reconstructed, which is why authorities cannot trace them. This is true of company-generated messages on Google’s home page, on YouTube (owned by Google), on Twitter, on Facebook and Instagram (owned by Meta), and other popular platforms. On YouTube, no records are kept of the sequences of videos shown to users, nor of that top video in the list, which is the “up-next” video that plays automatically unless the user selects a different video. On Twitter, company-generated tweets show only in the list you see when you first sign on; you can’t look at the tweets they showed you the last time you signed on.

And even though TMs can have a large impact on people’s opinions and votes (see below), virtually no one is aware that these messages are sent to some people and not others; without a large passive monitoring system in place that captures ephemeral content [51–53], no one can be certain that the manipulation even took place. Although some ephemeral political content was indeed being captured in the weeks leading up to the 2016 Presidential election [2,51], no one, to our knowledge, was tracking targeted messages sent by Facebook. Did Mr. Zuckerberg send out that go-vote reminder to Clinton supporters on Election Day? Unless he or a whistleblower comes forward to inform us, we will never know.

Other forms of online influence exist, of course, such as the influence exerted by thousands of bots launched by a secret organization in Russia to interfere with elections in the US [53–55], or micro-targeted ads posted by the now defunct company Cambridge Analytica in 2016 [56]. But manipulations like these–although occurring on our high-tech internet–are actually traditional in nature and are not, generally speaking, a threat to democracy. If Russian hackers launch a large number of anti-Biden bots, Biden’s party or another group of hackers could, in theory, launch its own bots to counter the Russian bots. This type of influence is very much like the influence exerted by billboards and television commercials: It is both visible and competitive [57], and as long as one has the resources, one can counteract it. Internet pioneers such as Tim Berners-Lee envisioned a future internet in which many thousands of relatively small entities would compete with each other for the attention of users [58], just as thousands of news media organizations have competed for people’s attention for a century or more. Unfortunately, as Berners-Lee himself has lamented in recent years, as the internet mushroomed in size, it became dominated (outside of mainland China) by “one search engine, one big social network, [and] one Twitter for microblogging” [59].

The dominance of such monopolies has put radically new and powerful means of influence into the hands of a small number of executives. For example, if Facebook–either through its main social media platform (S1 Fig) or through its subsidiary, Instagram (S2 Fig)–occasionally sends its users reminders to vote or reminders to register to vote, how would we know if these messages were being sent to all of its users or just to the members of one political party? The same could be said of Twitter, which currently inserts company-originated messages after every five or six tweets in people’s Twitter feeds, and of Google, which has been praised for including large “go-vote” messages on its home page on election days (S3-S5 Figs) [49,60]. If messages of this sort were being targeted to certain groups, unless a whistleblower came forward or a large-scale monitoring system was in place, we would not know, and, as we have noted, we would have no way to counter the manipulation.

Second, the term “digital gerrymander” already has a legitimate meaning in the social sciences. It refers to the use of computers to calculate optimal boundaries for voting districts [61,62]. Typically, this means boundaries that will virtually guarantee that one political party always wins. Computer modeling could also be used, of course, to guarantee maximum *fairness* in political redistricting, but that would rarely serve the interests of the people in power, and they are usually the people in charge of redistricting [63].

Third, the use of TMs for political purposes is just the tip of a very large iceberg. One immensely large class of TMs–targeted *advertisements*–impacts the purchases of millions of people every day. Nearly all of Facebook’s income comes from the fees companies pay to send their advertising content to targeted groups–people who appear, based on their Facebook profile and their most recent Facebook postings–to be highly likely to buy specific products from those companies. Because–at least in theory–any company can pay for that kind of advertising, it is inherently competitive and therefore no threat to consumers. But what if Facebook–in other words, the advertising *platform*–decided to ban certain ads or advertisers, or, more ominously, to throttle one company’s ads so that they often failed to reach the targeted audience? Again, without independent passive monitoring systems in place to capture the ephemeral content that actually reaches users, the manipulation of ads by platforms like Facebook and Amazon would be impossible to detect [2,51, cf. 64,65].

Fourth, targeted messaging–especially the messaging controlled by the large tech platforms themselves–can, in theory, influence almost any kind of thinking or behavior, not just political thinking or purchases. Targeted messages were certainly in wide use long before the internet was invented. Consequential messages have been delivered to specific groups of people on cigarette packs, condom boxes, pill containers–even on flimsy pieces of plastic used by cleaning companies to protect freshly cleaned clothes–and research has demonstrated the effectiveness of such messages, especially with certain populations [66,67]. The particular power that biased online messages have to alter thinking and behavior has also been demonstrated [68–70]. This is why we have set about trying to understand and quantify some aspects of the broader mechanism: Specifically, what happens when consequential messages are sent to one group and not another? How far apart can one push the groups? Will salient, high-contrast messages–that is, messages that stand out from a background–have a larger impact than subtle, low-contrast messages? Will the impact of a message increase if it is displayed multiple times? Can a single TM have a significant effect on people’s opinions and voting preferences? Do TMs on different platforms have comparable effects?

We will answer these questions in the experiments described herein. All four experiments employed procedures that were randomized, controlled, and double-blind, with all substantive content (such as the names and biographies of political candidates) counterbalanced to eliminate possible order effects.

## 2. Experiment 1: The impact of five low-contrast, verified, targeted messages on opinions and voting preferences

**Fig 1:**
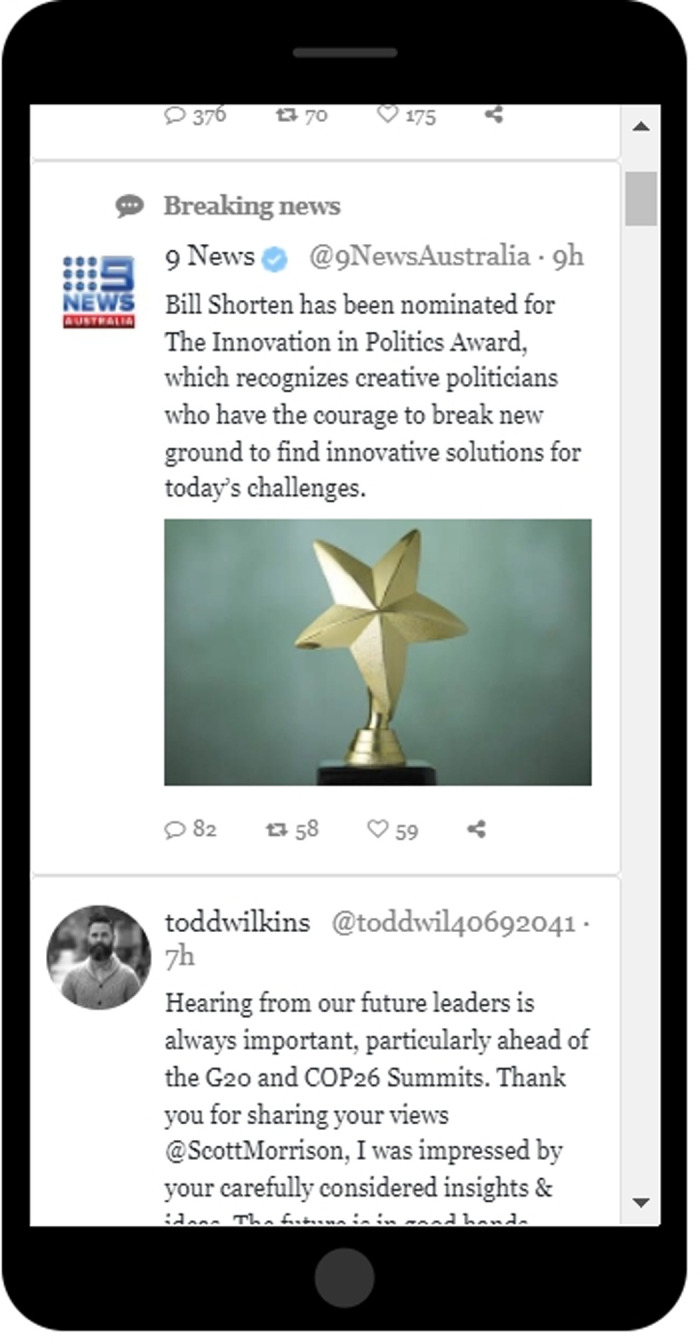
A screenshot showing an image of the first and second tweets in the Twitter feed employed in Experiment 1. The first tweet was a targeted message coming presumably from the Twitter company itself, in this case containing positive information about Bill Shorten. It would thus have been shown to study participants in the Pro-Shorten bias group. Its format was low-contrast (white background, with a black “Breaking News” headline) and included a blue checkmark, signifying verification. The second tweet in the image was an organic tweet sent by a fictitious user.

In our first experiment, we used a simulated Twitter feed to determine whether low-contrast, verified, targeted tweets could be used to shift the opinions and voting preferences of undecided voters. The appearance of these tweets closely matched that of the TMs the Twitter company currently sends to its users: (a) Our 5 TMs had a white background, just as our 30 organic tweets did. (b) The brief headline before the textual content read “Breaking News” in a black font. (c) The blue checkmark (signifying that Twitter had somehow “verified” the source of the tweet) was present on each TM, just as it is, at this writing, on all Twitter TMs (Fig 1). In order to assure that our participants would be “undecided,” we asked our US participants to express their views about two political candidates who ran for prime minister of Australia in 2019 [cf. 1].

### 2.1 Methods

#### 2.1.1 Ethics statement

The federally registered Institutional Review Board (IRB) of the sponsoring institution (American Institute for Behavioral Research and Technology) approved this study with exempt status under HHS rules because (a) the anonymity of participants was preserved and (b) the risk to participants was minimal. The IRB is registered with OHRP under number IRB00009303, and the Federalwide Assurance number for the IRB is FWA00021545. Informed written consent was obtained for all four experiments as specified in the Procedure section of Experiment 1.

#### 2.1.2 Participants

After cleaning, our participant sample for this experiment consisted of 533 eligible US voters recruited through the Amazon Mechanical Turk (MTurk) subject pool [71]. To avoid the growing problem with bots on MTurk, all participants were first screened and confirmed to be human by Cloud Research, a market research company. During the cleaning process, we removed participants who reported an English fluency level below 6 on a 10-point scale, where 1 was labeled “Not fluent” and 10 was labeled “Highly fluent.” We also removed participants who had reported a level of familiarity exceeding 3 on a 10-point scale with respect to either of the two political candidates referred to in the experiment, where 1 was labeled “Not familiar at all” and 10 was labeled “Very familiar.”

Our participants were demographically diverse in gender, age, race, and ethnicity, level of education completed, employment, income, and political leaning. See S1 Table for detailed demographic information for Experiments 1 through 4. The mean familiarity level for our first candidate, Scott Morrison, was 1.13, and the mean familiarity level for our second candidate, Bill Shorten, was 1.05.

#### 2.1.3 Procedure

Each session began with two screening questions. Participants could continue only if they said they were eligible to vote in the US and said no to the question, “Do you know a lot about politics in Australia?” They were then given basic instructions about the experiment, given information about how they could contact the experimenters with any questions or concerns they might have, and asked, in accordance with HHS rules, for their consent to participate. Participants were then asked a series of demographic questions, including questions about their political leanings, and then asked, on 10-point scales from “Not familiar at all” to “Very familiar,” how familiar they were with each of two Australian political candidates: Scott Morrison and Bill Shorten, as we noted above.

Participants were then given a short paragraph about each candidate (see S1 Text in Supporting Information for the full paragraphs), each about 120 words in length. Participants were next asked three opinion questions about each candidate: one regarding their overall impression of the candidate, one regarding how likeable they found the candidate, and one regarding how much they trusted the candidate. They were then asked, on an 11-point scale with values ranging from 5 to 0 to 5, which candidate they would be likely to vote for if they “had to vote today.” Finally, they were asked which candidate they would vote for if they “had to vote right now” (forced choice).

Participants were now given a task to complete: They would be given an opportunity to scroll through a series of tweets in order to gather information to help them decide which of the two candidates “will do a better job of protecting Australia from foreign threats.” They were instructed to scroll through “all the tweets” before making up their minds. See S2 Text for the complete instructions.

On the next screen, participants saw a mobile-phone image displaying a series of tweets (Fig 1). They could scroll through the Twitter feed either by dragging the scroll indicator on the scroll bar (right side of image) up or down, or by rotating the wheel on their mouse. For each participant, the maximum distance they scrolled downward through the Twitter feed was recorded as a percentage of the total distance.

Participants had been randomly assigned to one of three groups: Pro-Shorten, Pro-Morrison, or Control. People in all three groups had access to the same randomized sequence of 30 tweets authored by 30 different fictitious people; all the tweets were composed by the experimenters. Five of the tweets portrayed Bill Shorten in a positive light as a protector of Australia; five portrayed Scott Morrison in this same light; and the other 20 tweets simply commented on various ways of protecting Australia without referring to the candidates. All contained the hashtag #protectAustralia.

For participants in the Pro-Shorten and Pro-Morrison groups, 5 more tweets were added to the original sequence of 30 tweets, so users in these two “bias groups” had access to 35 tweets in all. In the context of this experiment, the five additional tweets should be considered TMs. These were messages presumably coming not from Twitter users but from the Twitter company itself. In real Twitter feeds, we estimate that the Twitter company typically inserts its own tweets roughly 20% of the time. Sometimes these messages are advertisements; sometimes they include links to breaking news stories; and, close to Election Day, they might include reminders to vote or to register to vote (see S6 and S7 Figs).

In the two bias groups, the TMs appeared in positions 2, 7, 12, 25, and 31 in the sequence of 35 tweets available in their Twitter feeds. The ordering and positions of the TMs were not varied. The only difference between the content seen by members of the Control group and members of the two bias groups was that people in the latter groups saw the five TMs, whereas people in the Control group did not. In addition, the only difference between the TMs seen by members of the Pro-Morrison bias group and members of the Pro-Shorten bias group was that people in the former group saw TMs favoring Morrison, whereas people in the latter group saw those same TMs with the names switched, so that they now favored Shorten.

For example, in the Pro-Morrison group, participants saw either strongly negative messages about Shorten such as “Bill Shorten charged with driving under the influence while vacationing in Adelaide,” or strongly positive messages about Morrison, such as “Scott Morrison awarded an honorary doctorate from the University of Melbourne, in recognition for his humanitarian efforts during the Australian wildfires.” As noted above, the TMs were identical in the pro-Shorten group, except that the candidate’s name was changed to his opponent’s name (see S3 Text for a complete list of TMs).

The Continue button in the lower-right corner of the web page was inactive for the first 30 seconds of the Twitter session, thus encouraging participants to spend some time reading tweets. If they clicked on the button before it was active, a message was displayed reading, “You have spent too little time reading this page. Please read more.” Also to encourage reading, a message appeared at the top of the page above the mobile phone image reading, “Scroll through the tweets below. You will need to spend some time viewing the tweets before you can continue to the next page.” A maximum of 5 minutes was allowed for examining the tweets in the Twitter feed.

On the surface, it might not be obvious how sending different tweets to people in different groups qualifies as targeted messaging. That we are indeed targeting our messages should be clearer if one imagines combining all of our participants into one large group. Now imagine that we divide the group up into three subgroups, perhaps based on certain demographic characteristics (such as income, gender, or political leaning). We now target the members of two of those subgroups with tweets favoring, say, one political candidate; we send no such tweets to the third subgroup. This is how targeted messaging works on any platform, and the message can contain almost any content: a prompt to vote or to register to vote, a reminder to buy one’s loved one a gift on Valentine’s Day, or an advertisement for throat lozenges. The message is targeted as long as it is deliberately being sent to one group and not another, and one knows the targeting has been effective if one can detect predictable changes in the behavior of the targeted group.

Following the Twitter experience, participants were again asked a series of questions. The first question was related to the task that had been assigned earlier. “Based on your Twitter search, which candidate, if either, do you think will do a better job of protecting Australia from foreign threats?” (11-point scale from 5 to 0 to 5). Following the “task” question, participants were again asked the six opinion questions and the two voting questions they had been asked before they saw the Twitter feed (see above).

Next, participants were asked whether any of the content they had seen in the Twitter feed “bothered” them in any way. They could reply yes or no, and then they could explain their answer by typing freely in a text box. This is a conservative way of determining whether people perceived any bias in the content they had seen–especially bias in the TMs that had been shown to people in the two bias groups. We could not ask people directly about their awareness of bias because leading questions of that sort often produce misleading answers [72].

The session concluded with general information about the goals of the research and about how people could withdraw their data from the study if they wished to do so. No participants chose to withdraw their data from any of the four experiments in the present study.

### 2.2 Results

Although participants were instructed to examine all the tweets in the Twitter feed (35 in the two bias groups, 30 in the control group), 29.0% of them did not comply, scrolling less than the full distance. Rather than discarding people with low scroll scores, we chose, for comparison purposes, to divide the sample into two groups: Low Compliance (maximum scroll values ≤ 50%) and High Compliance (maximum scroll values > 50%).

We call the shift in voting preferences “Vote Manipulation Power,” or VMP, which is the post-manipulation increase of people in the bias groups (expressed as a percentage increase) who said they would vote for the favored candidate [1]. For details about how VMP is calculated, see S4 Text. In the High Compliance group in Experiment 1, the VMP–the shift in voting preferences toward the favored candidate–was 87.0%, which is larger than any VMPs our team has ever found in SEME experiments [1]. A shift this large can, in theory, turn a 50/50 split among undecided voters into more than a 90/10 split (see S4 Text). The shift in the Low Compliance group–although smaller–was still substantial (Table 1).

**Table 1:** Experiment 1: VMPs by compliance level.

Compliance Level	Max Scroll	Total *n*	Bias Groups *n*	Bias Groups Scroll % Mean (*SD*)	VMP (%)	McNemar’s Test *X*^*2*^	*p*
**High**	> 50	434	287	93.5 (12.9)	87.0	103.14	< 0.001
**Low**	≤ 50	66	49	34.6 (11.3)	59.3	14.22	< 0.001

In the High Compliance group, answers to all six opinion questions shifted significantly in the direction predicted by the bias; in the Low Compliance group, answers to five of those six questions shifted significantly in that direction (Table 2), with the opinions shifting farther in the High Compliance group. Finally, the voting preferences as expressed on the 11-point opinion scale also shifted significantly and substantially in the predicted direction (see Table 3, where the data have been corrected for counterbalancing and candidate so that larger positive values indicate greater preference for the favored candidate).

**Table 2:** Experiment 1: Pre- and post-manipulation opinion ratings of candidates.

Compliance Level		Favored Candidate Mean (SD)			Non-Favored Candidate Mean (SD)			
		Pre	Post	Diff	Pre	Post	Diff	*z* ^†^
**High**	**Impression**	7.06 (1.79)	7.74 (1.98)	0.68	7.04 (1.79)	3.94 (2.03)	-3.10	-12.8***
	**Trust**	6.18 (1.98)	7.08 (2.06)	0.90	6.11 (1.92)	3.73 (2.16)	-2.38	-12.5***
	**Likeability**	7.07 (1.80)	7.51 (2.00)	0.44	6.93 (1.80)	4.17 (2.24)	-2.76	-12.0***
**Low**	**Impression**	7.10 (2.03)	7.55 (2.25)	0.45	7.02 (2.17)	4.02 (2.20)	-3.00	-5.06***
	**Trust**	5.76 (2.33)	6.63 (2.72)	0.87	5.53 (2.36)	3.53 (2.20)	-2.00	-4.81***
	**Likeability**	7.31 (1.84)	7.27 (2.18)	-0.04	7.08 (2.06)	4.04 (1.95)	-3.04	-4.89***

^†^z values represent Wilcoxon signed ranks test comparing post-minus-pre ratings for the favored candidate to the post-minus-pre ratings for the non-favored candidate.

***p < 0.001.

**Table 3:** Experiment 1: Pre- and post-manipulation mean voting preferences on 11-point scale (corrected so that positive values indicate preference for the favored candidate).

Compliance Level	Bias Groups *n*	Pre Voting Preference on 11-Point Scale (*SD*)	Post Voting Preference on 11-Point Scale (*SD*)	Mean Difference	*z*	*p*
**High**	287	-0.20 (2.77)	2.64 (2.43)	2.84	-12.07	< 0.001
**Low**	49	-0.04 (2.32)	2.73 (2.46)	2.77	-5.29	< 0.001

In the bias groups, only seven participants (out of 336, 2.1%) expressed concerns about possible bias in the content of the tweets; whereas 113 of these individuals (33.6%) commented specifically on the damaging (but never the positive) information in the biased TMs. Comments such as, “Read that Bill Shorten spent tax payer money, arrested for DUI and had an affair” and “Scott Morrison displayed a lot of bad judgment in his personal life (affairs, DUI arrests, etc.), which made me feel he was untrustworthy,” were common. People’s focus on the negative content in the TMs is addressed in Experiment 4 below, as well as in our Discussion section.

As we noted, Twitter’s TMs look almost exactly like the organic tweets of Twitter users. The main feature that consistently distinguishes the company’s TMs from most organic tweets is that their TMs all include the prestigious blue checkmark. In Experiment 2, we attempted to replicate our findings from Experiment 1 while omitting the blue checkmarks from our TMs.

## 3. Experiment 2: The impact of five low-contrast, non-verified targeted messages on opinions and voting preferences

### 3.1 Methods

#### 3.1.1 Participants

After cleaning, our participant sample for this experiment consisted of a new group of 532 eligible US voters recruited through the MTurk subject pool, screened once again by Cloud Research (see above). The cleaning procedure was identical to that of Experiment 1. Once again, the group was demographically diverse. See S1 Table for details about demographic characteristics. The mean familiarity level for our first candidate, Scott Morrison, was 1.10, and the mean familiarity level for our second candidate, Bill Shorten, was 1.04.

#### 3.1.2 Procedure

**Fig 2:**
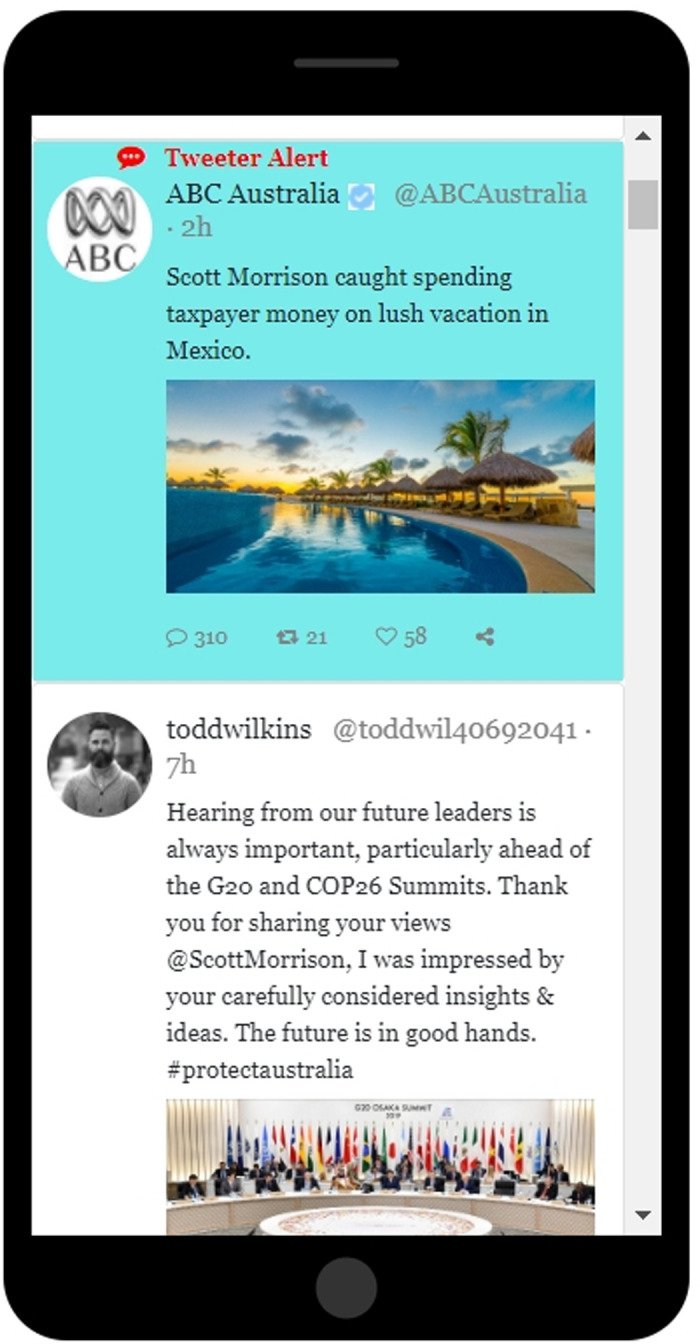
A screenshot showing an image of the second and third tweets in the Twitter feed employed in Experiment 3. The second tweet (top tweet in the image above) was a targeted message coming presumably from the Twitter company itself, in this case containing negative information about Scott Morrison. It would thus have been shown to study participants in the Pro-Shorten bias group. Its format was high-contrast (blue background, with a red “Tweeter Alert” headline).

The procedure in Experiment 2 was identical to that of Experiment 1, with one exception: The blue checkmark was *absent* on our TMs (Fig 2). Since that feature consistently distinguishes Twitter’s TMs from most organic tweets, we sought to determine whether the absence of this feature might reduce the impact of TMs on opinions and voting preferences.

### 3.2 Results

As expected, although the vote shifts were still quite large in both the Low and High Compliance groups, VMP values dropped substantially when the blue checks were absent (Table 4) (VMP_Expt1High_ = 87.0, VMP_Expt2High_ = 61.7, *z* = 8.58, *p* < 0.001).

**Table 4:** Experiment 2: VMPs by compliance level.

Compliance Level	Total *n*	Bias Groups *n*	Bias Groups Scroll % Mean (*SD*)	VMP (%)	McNemar’s Test *X*^*2*^	*p*
**High**	447	322	94.5 (12.3)	61.7	97.20	< 0.001
**Low**	61	48	34.1 (10.1)	44.4	10.29	< 0.01

Once again, opinions shifted in the predicted directions in all groups (Table 5), and so did mean voting preferences as expressed on the 11-point scale (Table 6). Even without the blue checkmarks on the TMs, participants also appeared to pay as much attention to them in Experiment 2 as in Experiment 1, with only 9 out of the 370 people in the bias groups (1.6%) raising concerns about possible bias in the content, and 115 of those people (31.1%) specifically mentioning the negative (but not the positive) things being said about the candidates in the TMs. The higher VMPs in Experiment 1 suggest that blue checkmarks add credibility to the content of the TMs, but the checkmarks do not seem to reduce the level of attention people are paying to them–or at least to the TMs with negative content.

**Table 5:** Experiment 2: Pre- and post-manipulation opinion ratings of candidates.

Compliance Level		Favored Candidate Mean (SD)			Non-Favored Candidate Mean (SD)			
		Pre	Post	Diff	Pre	Post	Diff	*z* ^†^
**High**	**Impression**	7.05 (1.76)	7.63 (1.86)	0.58	7.00 (1.74)	3.90 (1.97)	-3.10	-13.7***
	**Trust**	6.04 (2.04)	6.94 (2.05)	0.90	5.94 (2.00)	3.57 (1.94)	-2.37	-13.5***
	**Likeability**	6.95 (1.82)	7.39 (1.83)	0.44	6.91 (1.70)	4.02 (1.97)	-2.89	-13.6***
**Low**	**Impression**	7.38 (1.54)	7.52 (1.89)	0.14	7.40 (1.75)	4.17 (2.22)	-3.23	-4.76***
	**Trust**	6.40 (1.83)	6.73 (2.24)	0.33	6.54 (1.89)	3.88 (2.30)	-2.66	-4.54***
	**Likeability**	7.13 (1.41)	7.23 (1.75)	0.10	7.17 (1.59)	4.38 (2.64)	-2.79	-4.35***

^†^z values represent Wilcoxon signed ranks test comparing post-minus-pre ratings for the favored candidate to the post-minus-pre ratings for the non-favored candidate.

***p < 0.001.

**Table 6:** Experiment 2: Pre- and post-manipulation mean voting preferences on 11-point scale (corrected so that positive values indicate preference for the favored candidate).

Compliance Level	Bias Groups *n*	Pre Voting Preference on 11-Point Scale (*SD*)	Post Voting Preference on 11-Point Scale (*SD*)	Mean Difference	*z*	*p*
**High**	322	0.20 (2.61)	2.64 (2.39)	2.44	-12.56	< 0.001
**Low**	48	0.12 (2.66)	2.06 (2.62)	1.94	-3.68	< 0.001

Could substantially boosting the salience of TMs in a Twitter feed increase their impact on people’s opinions and voting preferences? We explore this question in Experiment 3.

## 4. Experiment 3: Impact of high-contrast, verified targeted messages on opinions and voting preferences

### 4.1 Methods

#### 4.1.1 Participants

After cleaning, our participant sample for this experiment consisted of a new group of 539 eligible US voters recruited through the MTurk subject pool, again screened by Cloud Research (see above). The cleaning procedure was identical to that of Experiment 1. Once again, the group was demographically diverse. See S1 Table for details about demographic characteristics. The mean familiarity level for our first candidate, Scott Morrison, was 1.14, and the mean familiarity level for our second candidate, Bill Shorten, was 1.04.

#### 4.1.2 Procedure

In Experiment 3 we deliberately altered the appearance of our TMs so that they would stand out. Specifically, we gave them blue backgrounds (instead of the usual white), and the message content was preceded by the words “Tweeter Alert” in a red font (Fig 2). The TMs also included Twitter’s iconic blue checkmarks. In all other respects, the procedure in Experiment 3 was identical to the procedure in Experiment 1.

### 4.2 Results

Because, generally speaking, increasing the salience of stimuli increases the attention they attract [73], one might expect that increasing the salience of the TMs would have increased their impact. The VMPs in Experiment 3, however, were significantly *lower* than the VMPs in Experiment 1, (Table 7) (VMP_Expt1High_ = 87.0, VMP_Expt3High_ = 81.1, *z* = 2.15, *p* < 0.05). Shifts in opinions (with one exception in the Low Compliance group) and voting preference as expressed on the 11-point scale also moved in the direction predicted by bias in the TMs (Tables 8 and 9), but, again, those shifts were lower than the ones we found in Experiment 1.

**Table 7:** Experiment 3: VMPs by compliance level.

Compliance Level	Total *n*	Bias Groups *n*	Bias Groups Scroll % Mean (*SD*)	VMP (%)	McNemar’s Test *X*^*2*^	*p*
**High**	446	287	95.3 (11.0)	81.1	114.29	< 0.001
**Low**	55	44	34.3 (9.6)	40.7	8.06	< 0.01

**Table 8:** Experiment 3: Pre- and post-manipulation opinion ratings of candidates.

Compliance Level		Favored Candidate Mean (SD)			Non-Favored Candidate Mean (SD)			
		Pre	Post	Diff	Pre	Post	Diff	*z* ^†^
**High**	**Impression**	6.92 (1.61)	5.61 (2.77)	-1.31	6.92 (1.71)	3.29 (2.11)	-3.63	-10.0***
	**Trust**	5.93 (1.84)	6.91 (2.19)	0.98	5.97 (1.92)	3.19 (1.91)	-2.78	-12.7***
	**Likeability**	6.79 (1.73)	7.43 (1.95)	0.64	6.74 (1.78)	3.65 (1.93)	-3.09	-13.3***
**Low**	**Impression**	7.75 (1.78)	5.48 (3.02)	-2.27	7.32 (1.88)	3.60 (2.53)	-3.72	-2.15***
	**Trust**	6.75 (1.92)	7.43 (2.22)	0.68	5.93 (2.27)	3.45 (2.28)	-2.48	-4.55***
	**Likeability**	7.55 (1.76)	7.84 (2.13)	0.29	7.39 (2.18)	4.09 (2.26)	-3.30	-4.82***

^†^z values represent Wilcoxon signed ranks test comparing post-minus-pre ratings for the favored candidate to the post-minus-pre ratings for the non-favored candidate.

***p < 0.001.

**Table 9:** Experiment 3: Pre- and post-manipulation mean voting preferences on 11-point scale (corrected so that positive values indicate preference for the favored candidate).

Compliance Level	Bias Groups *n*	Pre Mean Voting Preferences on 11-Point Scale (SD)	Post Mean Voting Preferences on 11-Point Scale (SD)	Mean Difference	*z*	*p*
**High**	287	0.03 (2.64)	2.95 (2.06)	2.92	-12.63	< 0.001
**Low**	44	0.95 (3.00)	2.77 (2.51)	1.82	-3.86	< 0.001

Once again, comments focused largely on the negative TMs– 98 out of 313 people in the bias groups (31.3%) mentioned negative TMs, compared to only 1 person who mentioned positive TMs and only 6 people (1.9%) who commented on possible bias in the tweets.

The findings from Experiments 1, 2, and 3 suggest that Twitter displays its TMs the way it does–in a manner that makes them difficult to distinguish from organic user tweets–to maximize their impact on users.

This leaves us with (at least) two intriguing questions: To what extent can a *single* TM shift opinions and voting preferences, if at all? And how much more impactful might a single *negative* TM be than a single *positive* TM? We address these questions in Experiment 4.

## 5. Experiment 4: Impact of a single low-contrast, verified targeted message on opinions and voting preferences

### 5.1 Methods

#### 5.1.1 Participants

After cleaning, our participant sample for this experiment consisted of a new group of 529 eligible US voters recruited through the MTurk subject pool and screened by Cloud Research. The cleaning procedure was identical to that of Experiment 1. Once again, the group was demographically diverse. See S1 Table for details about demographic characteristics.

#### 5.1.2 Procedure

The procedure in Experiment 4 was identical to that of Experiment 1, except that only one TM appeared in the Twitter feed. It appeared in position 2 for each bias group, and the blue checkmark was *present* in the TM.

Given the obvious preoccupation that participants had with negative TM content in Experiments 1 through 3, in Experiment 4 we looked at how positive and negative TMs impacted participants in the bias groups. Because people saw only one TM in this experiment, it was well suited for comparing the impact of positive and negative TMs. In the Pro-Morrison group, the TM could either be a pro-Morrison tweet (content: “Scott Morrison awarded an honorary doctorate from the University of Melbourne, in recognition for his humanitarian efforts during the Australian wildfires”) or an anti-Shorten tweet (content: “Bill Shorten charged with driving under the influence while vacationing in Adelaide”). In the Pro-Shorten group, the TM could either be a pro-Shorten tweet (content: “Bill Shorten has been nominated for The Innovation in Politics Award, which recognizes creative politicians who have the courage to break new ground to find innovative solutions for today’s challenges”) or an anti-Morrison tweet (content: “Scott Morrison caught spending taxpayer money on lush vacation in Mexico”); again, one or the other appeared at random.

### 5.2 Results

At first glance, the pattern of VMPs we found in Experiment 4 looks surprising (Table 10). In Experiments 1 to 3, the VMPs in the High Compliance groups were always substantially larger than the VMPs in the Low Compliance group. In Experiment 4 we found the opposite pattern, most likely because people in the Low Compliance group saw, on average, only 36.2% of the tweets following the TM in position 2, whereas people in the High Compliance group saw, on average, 97.4% of the tweets following that TM. Exposure to a large number of relatively bland tweets following a biased TM apparently dilutes the power of that TM. Finally, once again, very few people claimed that they saw any bias in the Twitter feed we showed them; only 3 out of the 399 people in the bias groups (0.75%) expressed concerns about possible bias in the content, and 76 of those people (19.0%) specifically mentioned the negative (but not the positive) things being said about the candidates in the TMs.

**Table 10:** Experiment 4: VMPs by compliance level.

Compliance Level	Total *n*	Bias Groups *n*	Bias Groups Scroll % Mean (*SD*)	VMP (%)	McNemar’s Test *X*^*2*^	*p*
**High**	445	356	97.4 (8.5)	32.4	31.54	< 0.001
**Low**	50	43	36.2 (11.2)	40.0	6.40	< 0.05

Breaking down the impact of positive TMs versus negative TMs on the VMPs in Experiment 4 confirms the enormous power that negative content has to alter people’s thinking (Table 11). The positive TMs had virtually no impact on VMPs in either the Low Compliance or High Compliance groups. The negative TMs, on the other hand, had a relatively large impact on High Compliance participants (VMP = 51.2%) and shifted *all* of the 17 Low Compliance participants (VMP = 100.0%). Only one of the 399 people in the bias groups expressed any concerns about possible bias in the tweets (0.003%), whereas 76 of these individuals (19.0%) specifically singled out the negative content of the single TM (regarding the candidate’s DUI conviction) as a reason for not supporting him. No participants mentioned the contents of the positive version of the TM (regarding the candidate receiving The Innovation in Politics Award) in their typed comments. The possibility of bias was mentioned somewhat more frequently in comments in Experiments 1 through 3, presumably because people in the bias groups in those experiments saw as many as five TMs that shared the same bias; in Experiment 4, people saw only one TM.

**Table 11:** Experiment 4: VMPs by type of TM (positive or negative).

Type of TM	Compliance Level	Bias Groups *n*	VMP (%)	McNemar’s Test *X*^*2*^	*p*
**Negative**	**High**	178	51.2	31.72	< 0.001
	**Low**	17	100.0	6.00	< 0.05
**Positive**	**High**	178	15.6	4.08	< 0.05
	**Low**	26	14.3	1.00	0.32 NS

Most opinion shifts in the bias groups in Experiment 4 occurred in the predicted direction (Table 12), but they were smaller than the shifts found in the earlier experiments, presumably because participants had less information on which to base their opinions. Changes in voting preferences as expressed on the 11-point scale also occurred in the predicted direction, but, again, they were smaller than in the previous experiments (Tables 13 and 14).

**Table 12:** Experiment 4: Pre- and post-manipulation opinion ratings of candidates.

			Favored Candidate Mean (SD)				Non-Favored Candidate Mean (SD)			
	Compliance		Pre	Post		Diff	Pre	Post	Diff	*z* ^†^
**Negative TM**										
	**High**	**Impression**	7.03 (1.92)	7.13 (1.92)		-0.10	7.11 (1.93)	5.39 (2.20)	1.72	-7.91***
		**Trust**	6.05 (1.80)	6.51 (2.01)		-0.46	6.19 (1.88)	5.03 (2.25)	1.16	-7.38***
		**Likeability**	6.72 (1.89)	6.94 (1.86)		-0.22	7.04 (1.86)	5.53 (2.13)	1.51	-7.99***
	**Low**	**Impression**	6.53 (1.62)	6.65 (1.93)		0.12	7.06 (1.85)	4.65 (1.90)	-2.41	-3.08**
		**Trust**	6.18 (2.27)	6.29 (1.40)		0.11	6.18 (2.19)	4.59 (2.29)	-1.59	-2.34*
		**Likeability**	6.88 (1.80)	6.47 (1.97)		-0.41	7.12 (1.62)	5.18 (2.53)	-1.94	-1.79 NS
**Positive TM**										
	**High**	**Impression**	7.31 (1.84)	7.26 (1.85)		0.05	7.17 (1.92)	7.05 (1.86)	0.12	-0.77 NS
		**Trust**	6.30 (1.95)	6.56 (1.97)		-0.26	6.22 (2.07)	6.39 (2.02)	-0.17	-0.85 NS
		**Likeability**	7.19 (1.87)	7.18 (1.93)		0.01	7.01 (1.91)	6.98 (1.88)	0.03	-0.59 NS
	**Low**	**Impression**	7.00 (2.21)	7.19 (2.32)		0.19	7.12 (1.93)	7.38 (1.92)	0.26	-0.29 NS
		**Trust**	6.04 (2.39)	6.23 (2.76)		0.19	6.12 (2.16)	6.50 (2.25)	0.38	-0.18 NS
		**Likeability**	6.85 (2.43)	6.81 (2.28)		-0.04	7.27 (1.93)	7.15 (1.87)	-0.12	-0.21 NS

^†^z values represent Wilcoxon signed ranks test comparing post-minus-pre ratings for the favored candidate to the post-minus-pre ratings for the non- favored candidate.

* *p* < 0.05

** *p* < 0.01

***p < 0.001.

**Table 13:** Experiment 4: Negative TM Pre- and post-manipulation mean voting preferences on 11-point scale (corrected so that positive values indicate preference for the favored candidate).

Compliance Level	*n*	Pre Voting Preference on 11-Point Scale (SD)	Post Voting Preference on 11-Point Scale (SD)	Mean Difference	*z*	*p*
**High**	178	-0.11 (2.85)	1.31 (2.88)	1.42	-6.42	< 0.001
**Low**	17	-0.47 (2.76)	1.41 (2.50)	0.64	-2.60	< 0.01

**Table 14:** Experiment 4: Positive TM pre- and post-manipulation mean voting preferences on 11-point scale (corrected so that positive values indicate preference for the favored candidate).

Compliance Level	*n*	Pre Voting Preference on 11-Point Scale (SD)	Post Voting Preference on 11-Point Scale (SD)	Mean Difference	*z*	*p*
**High**	178	0.21 (2.77)	0.74 (2.78)	0.53	-2.58	= 0.01
**Low**	26	0.58 (2.86)	0.65 (3.03)	0.07	-0.20	= 0.84

Experiment 4 suggests that a single biased TM in a Twitter feed can impact people’s decision making, at least as it pertains to political candidates running for office.

## 6. Discussion

Recent news about the Twitter company is relevant to our research findings. According to an August 23rd, 2022, investigative story in the *Washington Post* [74], “an explosive whistleblower complaint” from Peter Zatko, former head of security at Twitter–an 84-page document filed simultaneously with the Securities and Exchange Commission, the Federal Trade Commission, and the Department of Justice [75]–Twitter had lax security that allowed false content to be posted easily by hackers, bots, foreign powers, and company employees. Regarding employees, the *Post* reported that “about half of Twitter’s roughly 7,000 full-time employees had wide access to the company’s internal software and that access was not closely monitored, giving them the ability to tap into sensitive data and alter how the service worked.” According to Zatko, Twitter algorithms also determined what content gets suppressed or “amplified” [76].

Given Elon Musk’s purchase of the company in October, 2022 [77] and his subsequent firing of most of Twitter’s employees, Zatko’s concerns about the security of the company’s operations might understate the nature of the problems that might be emerging in a new and relatively unstable version of the company. Given the apparent power that tweets–especially tweets containing negative content–can have on opinions and voting preferences–we believe that Twitter’s operations should be examined closely not only by Twitter’s corporate leaders, but also by government officials and public policy makers in countries worldwide. Twitter currently has 480 million daily users, and it serves as an official platform for world leaders, government agencies, news services, and thousands of companies and organizations; even Pope Francis has a Twitter account. All those Twitter feeds are vulnerable to hacking and hijacking, according to Zatko’s complaint, which contains examples of such interference.

Our experiments suggest that TME is a remarkably large effect, especially when Twitter itself sends people sensational tweets that have certain visual properties (Experiment 1): tweets with white backgrounds (matching the backgrounds of organic tweets), a brief headline (such as “Breaking News”), and Twitter’s trademark blue checkmark. Experiment 1 yielded a VMP of 87%, with only 2.1% of the participants in the two bias groups expressing any concerns about possible bias in the Twitter feed we showed them. That VMP shift means that in a group of 1,000 undecided voters–split, by definition, 500/500 before exposure to a biased Twitter feed–after viewing that feed, the split will now be 65/935, which means that interacting with the Twitter feed changed a win margin of 0% to a win margin of 87% among vulnerable voters. That shift could occur, in theory, with nearly 98% of the people in such a group having no idea they were manipulated.

On its face, a shift that big might seem impossible. In the real world, certainly, people are being influenced by many sources of information, not just by Twitter, and we currently have no reason to believe that Twitter’s content is systematically biased to support just one candidate or political party. But our experiments show the *potential* that Twitter feeds have to shift opinions and votes. Twitter is a private company that is not accountable to the public, and no laws or regulations exist at this writing that would in any way restrict Twitter’s ability to send highly biased content to users. Indeed, some people have claimed that Twitter’s content already shows significant political bias at times [cf. 76]. Trump supporters cried foul, for example, when Twitter permanently shut down the President’s Twitter account just after the January 6, 2021 insurrection in Washington, D.C. [78], and objections were raised when Twitter apparently suppressed news stories related to content found on Hunter Biden’s laptop computer in October 2020 [79]. In this case, some of the facts about the laptop originally reported by the *New York Post* on October 14, 2020 were subsequently confirmed by both the *New York Times* and the *Washington Post* [80, cf. 81]. That Twitter content might show political bias should surprise no one given that, according to OpenSecrets.org, more than 96% of donations from Twitter and its employees in recent years have gone to one political party [43].

No experiments can show that a source of influence like TME is actually being used. Since 2016, however, our team has been building increasingly larger and more sophisticated systems that capture the ephemeral content being shown to users by Google, YouTube, Bing, Yahoo, and other companies [51,52,82]. In 2020, we preserved and analyzed more than 1.5 million online ephemeral experiences that would normally have been lost [52]. In 2022, we preserved more than 2.4 million online ephemeral experiences related to the US midterm elections, including, this time around, content from Twitter which we are currently analyzing.

We acknowledge that if, at some point, we detect political or other bias in Twitter feeds being displayed to certain groups, that will still tell us nothing about the origin of such bias. Bias in ephemeral content can be programmed deliberately [83], generated by unconscious bias on the part of programmers [84], or generated by user behavior [85]. No matter what the original of such bias, given the apparent power it has to shift opinions and voting preferences, we believe that if large-scale bias is ultimately found to exist in actual Twitter feeds, this is an issue that Twitter executives and government officials will need to examine. Otherwise, extreme bias–especially bias targeted toward certain groups–could easily undermine the integrity of the free-and-fair election. Moreover, if monitoring systems are not in place to preserve ephemeral content such as Twitter feeds, democracy might be undermined without the electorate knowing. Based on Mr. Zatko’s recent revelations [74,75], along with documented cases in which Twitter content has been hacked by bad actors [86,87], it now appears that extreme bias in Twitter content can be introduced fairly easily by agents of foreign powers, by aggressive Twitter employees, or even by mischievous teenagers [74,75].

### 6.1 Limitations and future research

We have restricted ourselves in this report to TME as it might impact users of Twitter, but targeted messages can also be sent to users of Google and other search engines (S3 and S5 Figs), to users of Instagram and Facebook (S1 and S2 Figs), and even to users of personal assistants such as Siri and Alexa [4]. On platforms such as Google, the home page of which is viewed more than 500 million times a day in the US, we are especially concerned about targeted messages that remind people to vote or to register to vote in an election (S3 and S5 Figs). If such reminders were sent mainly or exclusively to members of one political party, they could presumably have a substantial partisan effect on voter turnout. We currently have research underway to help us understand and quantify the impact that TME might have on Google and other online platforms.

We are also concerned about the possibility that a number of major US tech companies all appear at the moment to share a similar political bias [88], and we are currently studying the impact of exposing people to similar or dissimilar bias experienced on more than one platform–research on what we call the Multiple Platform Effect (MPE). We have also expanded our research program to look at how new sources of influence made possible by the internet are affecting children.

Our findings in the present study should not be overinterpreted. We have shown, with a sample of 2,133 eligible US voters that biased, targeted tweets can shift opinions and voting preferences in predictable ways with only a small percentage of people showing any awareness that they have been manipulated. The effect proved to be especially large when the content of such tweets was derogatory (content that linguists might call “low-valence and high-arousal” [89]. But our participants were not real voters in the middle of real elections. Rather, they were US research subjects who had indicated that they were unfamiliar with two candidates who ran for Prime Minister of Australia in 2019. SEME has been shown to impact real voters in a real election [1], but TME has not yet been tested that way.

In a real election people are being subjected to dozens, if not hundreds of different sources of influence that might affect their voting decisions. Other sources of impact could presumably override the impact of biased tweets, and yet there are still, we believe, three reasons why we should be concerned about TME in general and corporation-generated biased tweets in particular. As we noted in our introduction, the bias in TMs is almost always invisible to people, which leads people, mistakenly, to believe that they have made up their own minds. Second, TMs are ephemeral, so unless permanent monitoring systems are in place, we will never know for sure how or even whether TMs are being used to affect people’s opinions and decisions. And third, TMs generated by large online monopolies are inherently noncompetitive; when Twitter, Facebook, or Google deploys biased TMs favoring one candidate, the opposing candidate has no way to counteract them. In other words, online TMs are a uniquely powerful new form of influence.

## Supporting information

S1 Fig Facebook vote reminder, screenshotted in Georgia, January 5, 2021. (DOCX)

S2 Fig Instagram vote reminder, screenshotted in Georgia January 5, 2021. (DOCX)

S3 Fig Google home page with vote reminder, 2020 Presidential election, screenshotted October 27, 2020. (DOCX)

S4 Fig Google home page with no vote reminder, 2020 Presidential election, screenshotted October 27, 2020. (DOCX)

S5 Fig Google home page with vote reminfer, 2020 Presidential election, screenshotted on Election Day, November 3, 2020. (DOCX)

S6 Fig Twitter home page with vote reminder, 2022 Midterm election, screenshotted November 7, 2022. (DOCX)

S7 Fig Twitter home page with a “You might like” promoted tweet containing a vote reminder, screenshotted November 8, 2022. (DOCX)

S8 Fig Example of a control tweet presented to all participants in Experiments 1–4. (DOCX)

S9 Fig Example of a strongly negative targeted message about Morrison with a blue checkmark, presented to participants in the Pro-Shorten group in Experiment 1. (DOCX)

S10 Fig Example of a strongly negative targeted message about Morrison without a blue checkmark, presented to participants in the Pro-Shorten group in Experiment 2. (DOCX)

S1 Table Demographics characteristics across Experiments 1 to 4. (DOCX)

S2 Table Experiment 1: Demographic analysis by educational attainment. (DOCX)

S3 Table Experiment 1: Demographic analysis by gender. (DOCX)

S4 Table Experiment 1: Demographic analysis by age. (DOCX)

S5 Table Experiment 1: Demographic analysis by race/ethnicity. (DOCX)

S6 Table Experiment 2: Demographic analysis by educational attainment. (DOCX)

S7 Table Experiment 2: Demographic analysis by gender. (DOCX)

S8 Table Experiment 2: Demographic analysis by age. (DOCX)

S9 Table Experiment 2: Demographic analysis by race/ethnicity. (DOCX)

S10 Table Experiment 3: Demographic analysis by educational attainment. (DOCX)

S11 Table Experiment 3: Demographic analysis by gender. (DOCX)

S12 Table Experiment 3: Demographic analysis by age. (DOCX)

S13 Table Experiment 3: Demographic analysis by race/ethnicity. (DOCX)

S14 Table Experiment 4: Demographic analysis by educational attainment. (DOCX)

S15 Table Experiment 4: Demographic analysis by gender. (DOCX)

S16 Table Experiment 4: Demographic analysis by age. (DOCX)

S17 Table Experiment 4: Demographic analysis by race/ethnicity. (DOCX)

S18 Table Experiment 1: Pre-and post-manipulation opinions by group. (DOCX)

S19 Table Experiment 2: Pre-and post-manipulation opinions by group. (DOCX)

S20 Table Experiment 3: Pre-and post-manipulation opinions by group. (DOCX)

S21 Table Experiment 4: Pre-and post-manipulation opinions by group. (DOCX)

S22 Table Experiments 1–3: Pre- and post-manipulation votes on 11-point scale (-5 to +5). (DOCX)

S23 Table Experiment 4: Pre- and post-manipulation vote on 11-point scale (-5 to +5). (DOCX)

S1 Text Experiment 1: Candidate biographies. (DOCX)

S2 Text Experiments 1 to 4: Instructions immediately preceding Twitter simulation. (DOCX)

S3 Text Experiments 1 to 4: Textual content and positions of the five targeted messages. (DOCX)

S4 Text Vote Manipulation Power (VMP) calculation. (DOCX)
