# Supplementary figures and images for: What would happen if twitter sent consequential messages to only a strategically important subset of users? A quantification of the Targeted Messaging Effect (TME)
Source: PLoS One. 2023 Jul 27;18(7):e0284495. doi: 10.1371/journal.pone.0284495 (PMC10374154; doi:10.1371/journal.pone.0284495)

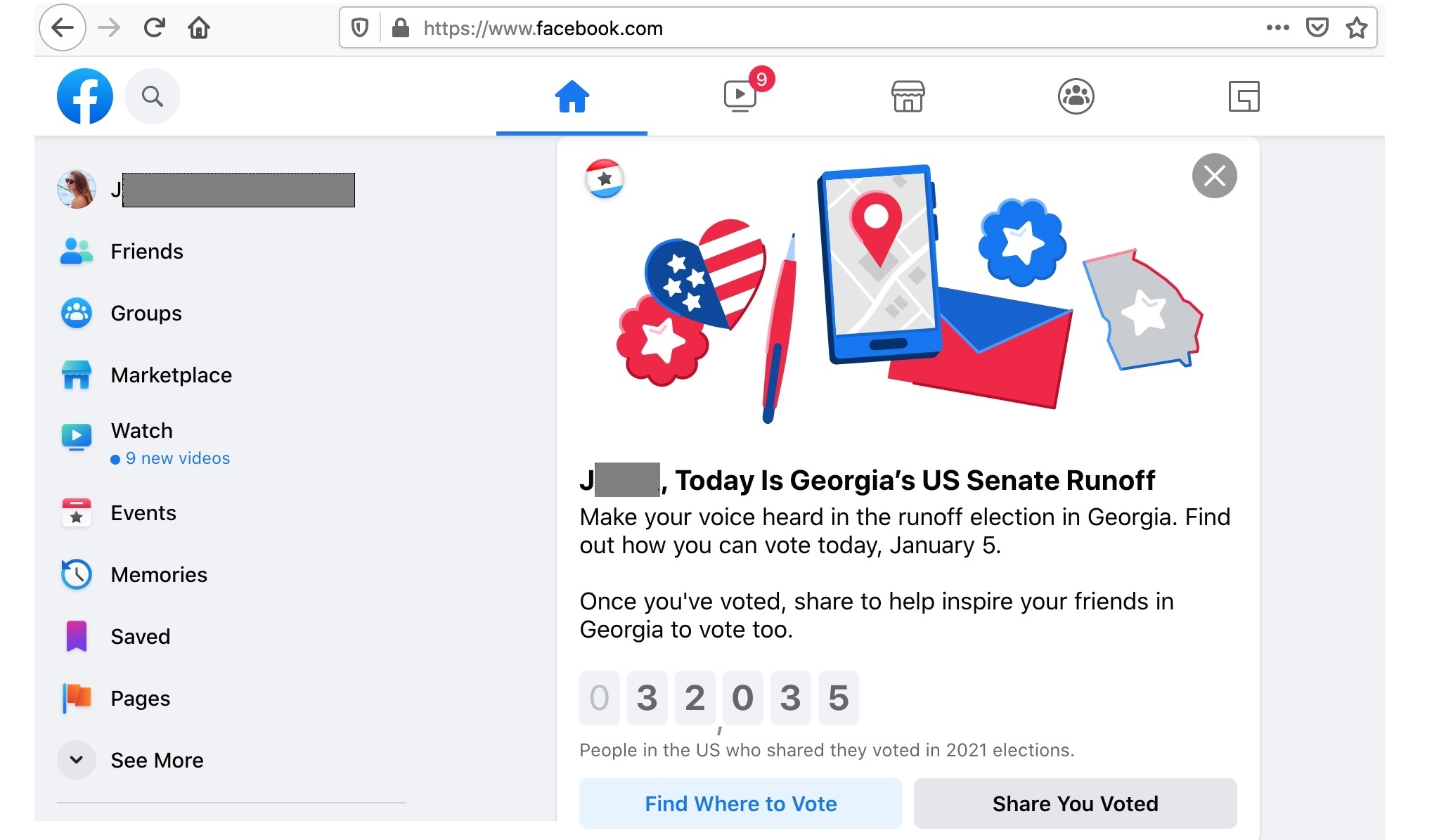


**S1 Fig. Facebook vote reminder, screenshotted in Georgia, January 5, 2021.**

Supplement: S1 Fig — (DOCX) [file pone.0284495.s001.docx]

**
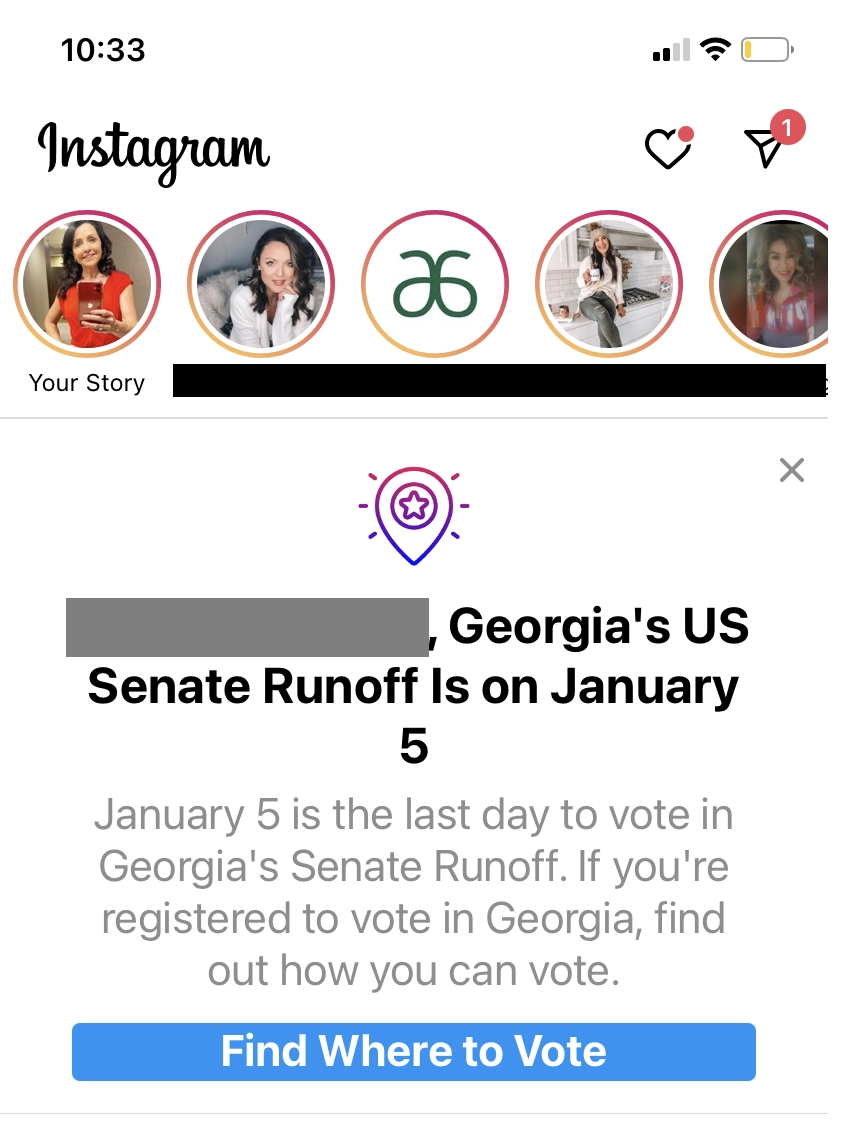
S2 Fig.** **Instagram vote reminder, screenshotted in Georgia January 5, 2021.**

Supplement: S2 Fig — (DOCX) [file pone.0284495.s002.docx]

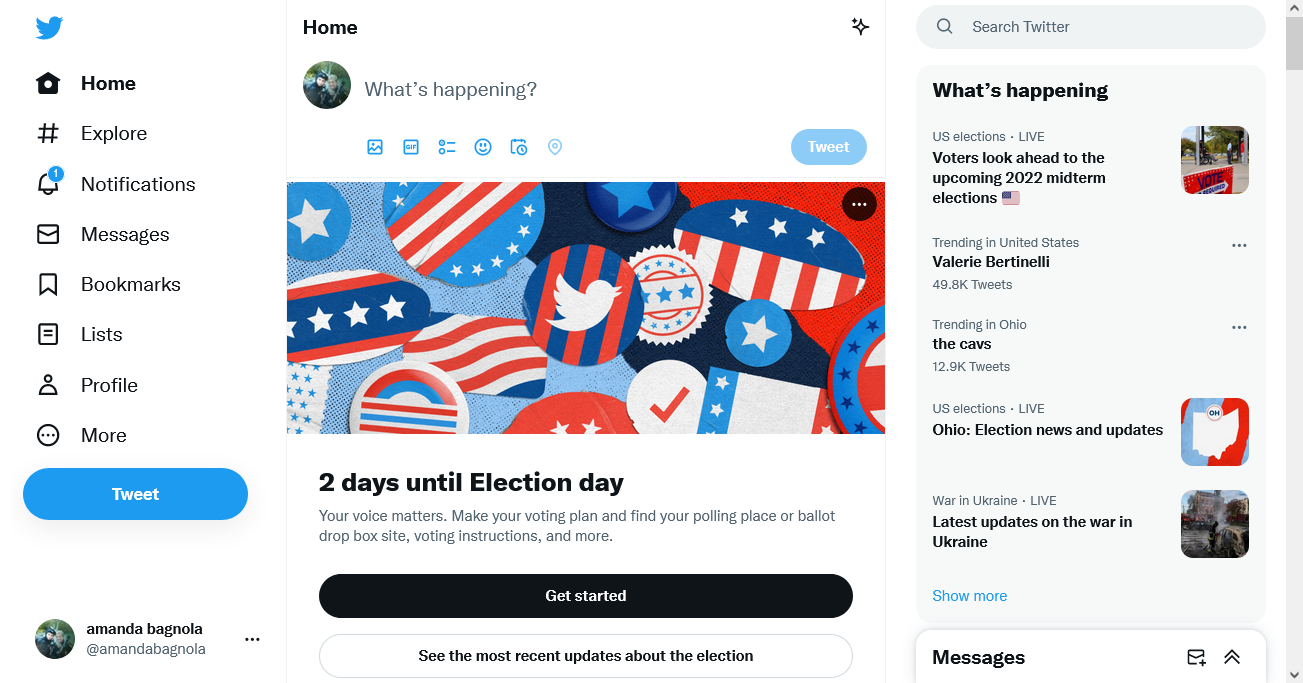


**S6 Fig. Twitter home page with vote reminder, 2022 Midterm elections, screenshotted November 7, 2022.**

Supplement: S6 Fig — (DOCX) [file pone.0284495.s006.docx]

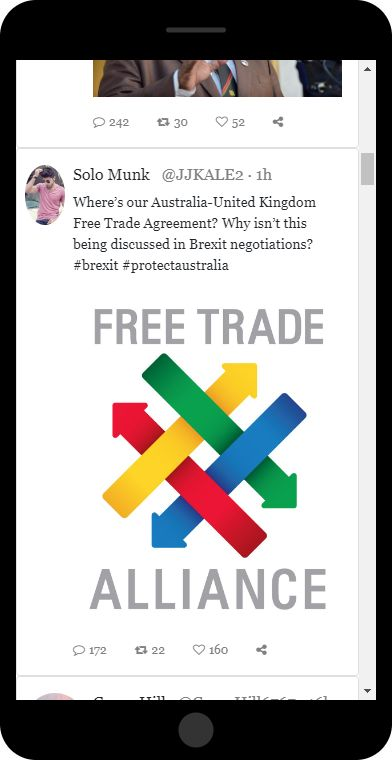


**S8 Fig. Example of a control tweet presented to all participants in Experiments 1-4.**

Supplement: S8 Fig — (DOCX) [file pone.0284495.s008.docx]
