# Supplementary material for: What would happen if twitter sent consequential messages to only a strategically important subset of users? A quantification of the Targeted Messaging Effect (TME)
Source: PLoS One. 2023 Jul 27;18(7):e0284495. doi: 10.1371/journal.pone.0284495 (PMC10374154; doi:10.1371/journal.pone.0284495)
Supplement: S3 Fig — (DOCX) [file pone.0284495.s003.docx]

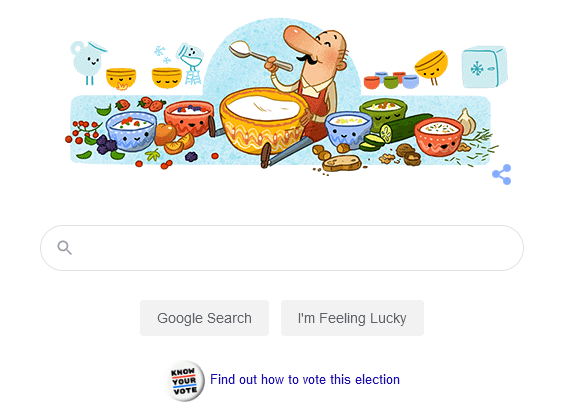


**S3 Fig. Google home page with vote reminder, 2020 Presidential election, screenshotted October 27, 2020.** Sent to a politically liberal field agent (see text).
