# Supplementary material for: What would happen if twitter sent consequential messages to only a strategically important subset of users? A quantification of the Targeted Messaging Effect (TME)
Source: PLoS One. 2023 Jul 27;18(7):e0284495. doi: 10.1371/journal.pone.0284495 (PMC10374154; doi:10.1371/journal.pone.0284495)
Supplement: S7 Fig — (DOCX) [file pone.0284495.s007.docx]

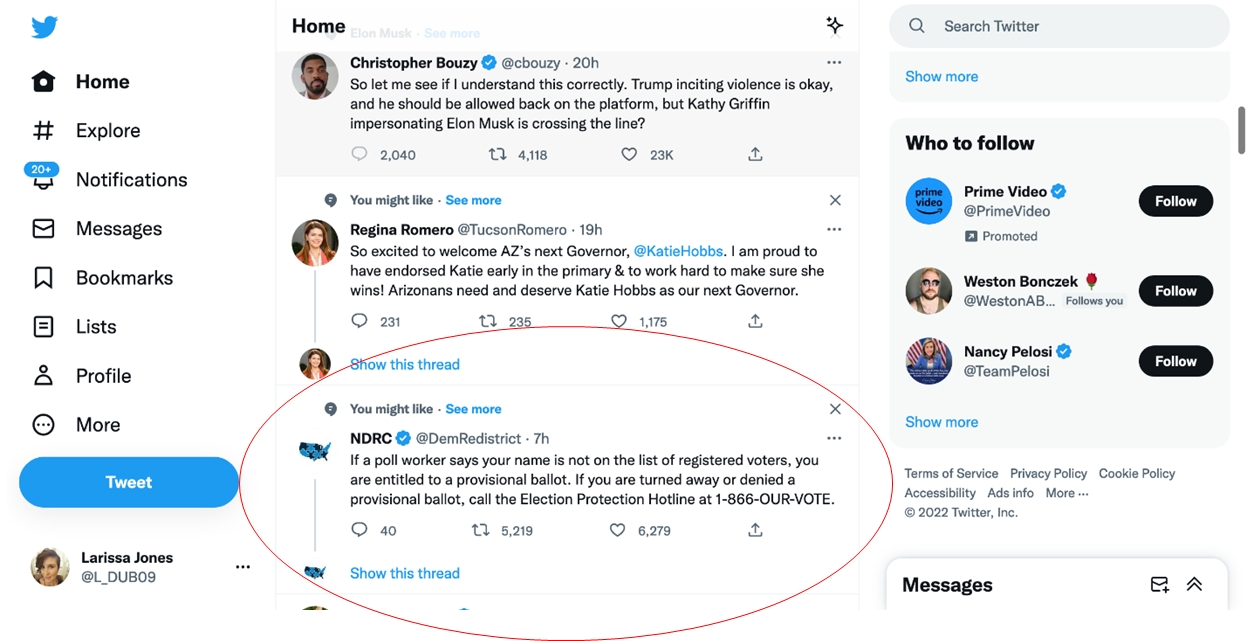


**S7 Fig. Twitter home page with a “You might like” promoted tweet containing a vote reminder, screenshotted November 8, 2022.** The authors added the red oval highlighting the relevant tweet.
