# Supplementary material for: What would happen if twitter sent consequential messages to only a strategically important subset of users? A quantification of the Targeted Messaging Effect (TME)
Source: PLoS One. 2023 Jul 27;18(7):e0284495. doi: 10.1371/journal.pone.0284495 (PMC10374154; doi:10.1371/journal.pone.0284495)
Supplement: S10 Fig — (DOCX) [file pone.0284495.s010.docx]

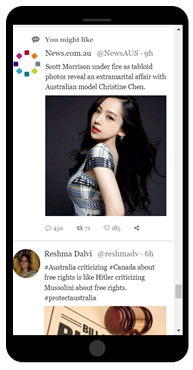


**S10 Fig. Example of a strongly negative targeted message about Morrison without a blue checkmark, presented to participants in the Pro-Shorten group in Experiment 2.**
