# Supplementary material for: What would happen if twitter sent consequential messages to only a strategically important subset of users? A quantification of the Targeted Messaging Effect (TME)
Source: PLoS One. 2023 Jul 27;18(7):e0284495. doi: 10.1371/journal.pone.0284495 (PMC10374154; doi:10.1371/journal.pone.0284495)
Supplement: S1 Table — (DOCX) [file pone.0284495.s011.docx]

|  | **Experiment 1**  **(*n* = 533)** | | | **Experiment 2**  **(*n* = 532)** | | | **Experiment 3**  **(*n* = 539)** | | **Experiment 4**  **(*n* = 529)** |
| --- | --- | --- | --- | --- | --- | --- | --- | --- | --- |
| **Mean Age (*SD*)** |  | |  | | | |  | |  |
|  | 38.6 (12.8) | | 38.4 (12.5) | | | | 38.7 (12.1) | | 37.0 (11.8) |
| **Gender (*n*)** |  | |  | | |  | | |  |
| **Male** | 208 (39.0%) | | 194 (36.4%) | | | 212 (39.3%) | | | 205 (38.8%) |
| **Female** | 323 (60.6%) | | 331 (62.2%) | | | 318 (59.0%) | | | 319 (60.3%) |
| **Other** | 0 (0.0%) | | 0 (0.0%) | | | 0 (0.0%) | | | 0 (0.0%) |
| **Unknown** | 2 (0.4%) | | 7 (1.32%) | | | 9 (1.7%) | | | 5 (0.9%) |
| **Political View (*n*)** | |  | | |  | | |  | |
| **Conservative** | 119 (22.3%) | | 116 (21.8%) | | | 137 (25.4%) | | | 105 (19.8%) |
| **Liberal** | 225 (42.2%) | | 217 (40.8%) | | | 249 (46.2%) | | | 228 (43.1%) |
| **Moderate** | 150 (28.1%) | | 163 (30.6%) | | | 121 (22.4%) | | | 171 (32.3%) |
| **None** | 28 (5.3%) | | 27 (5.08%) | | | 23 (4.27%) | | | 22 (4.16%) |
| **Other** | 11 (2.1%) | | 9 (1.69%) | | | 9 (1.67%) | | | 3 (0.56%) |
| **Unknown** | 0 (0.0%) | | 0 (0.0%) | | | 0 (0.0%) | | | 0 (0.0%) |
| **Voter Status** |  | |  | | |  | | |  |
| **Decided** | 0 (0.0%) | | 0 (0.0%) | | | 0 (0.0%) | | | 0 (0.0%) |
| **Undecided** | 533 (100%) | | 532 (100%) | | | 539 (100%) | | | 529 (100%) |
| **Unknown** | 0 (0.0%) | | 0 (0.0%) | | | 0 (0.0%) | | | 0 (0.0%) |
| **Fluency (SD)** |  | |  | | |  | | |  |
|  | 9.94 (0.3) | | 9.9 (0.3) | | | 10.0 (0.2) | | | 9.9 (0.3) |

**S1 Table. Demographics characteristics across Experiments 1 to 4.**
