# Supplementary material for: What would happen if twitter sent consequential messages to only a strategically important subset of users? A quantification of the Targeted Messaging Effect (TME)
Source: PLoS One. 2023 Jul 27;18(7):e0284495. doi: 10.1371/journal.pone.0284495 (PMC10374154; doi:10.1371/journal.pone.0284495)
Supplement: S2 Table — (DOCX) [file pone.0284495.s012.docx]

**S2 Table. Experiment 1: Demographic analysis by educational attainment.**

| **Condition** |  | ***n*** | **VMP (%)** | **Mean Search Time (sec) (SD)** | **Mean Scroll-Max Percentage (SD)** |
| --- | --- | --- | --- | --- | --- |
| **Bias Groups** | **≥ Bachelors** | 220 | 85.3% | 173.0 (109.7) | 85.8 (23.2) |
|  | **< Bachelors** | 138 | 75.4% | 217.4 (195.3) | 84.3 (26.4) |
|  | **Change (%)** | - | +11.6% | -25.7% | +1.8% |
|  | **Statistic** | *-* | *z* = 2.35 | t(192) = -2.44 | t(334) = 0.55 |
|  | ***p*** | - | < 0.05 | < 0.05 | = 0.58 NS |
| **Control Group** | **≥ Bachelors** | 94 | - | 193.0 (163.0) | 92.0 (19.4) |
|  | **< Bachelors** | 81 | - | 176.5 (110.9) | 87.0 (26.1) |
|  | **Change (%)** | - | - | 8.6% | 5.4% |
|  | **Statistic** | *-* | *-* | t(173) = 0.77 | t(134) = 1.37 |
|  | ***p*** | - | - | = 0.44 NS | = 0.17 NS |
