# Supplementary material for: What would happen if twitter sent consequential messages to only a strategically important subset of users? A quantification of the Targeted Messaging Effect (TME)
Source: PLoS One. 2023 Jul 27;18(7):e0284495. doi: 10.1371/journal.pone.0284495 (PMC10374154; doi:10.1371/journal.pone.0284495)
Supplement: S3 Table — (DOCX) [file pone.0284495.s013.docx]

**S3 Table. Experiment 1: Demographic analysis by gender.**

| **Condition** |  | ***n*** | **VMP (%)** | **Mean Search Time (sec) (SD)** | **Mean Scroll-Max Percentage (SD)** |
| --- | --- | --- | --- | --- | --- |
| **Bias Groups** | **Male** | 134 | 85.2% | 204.8 (185.7) | 86.3 (24.6) |
|  | **Female** | 223 | 80.0% | 181.9 (123.3) | 84.6 (24.4) |
|  | **Change (%)** | - | +6.1% | +11.2% | +2.0% |
|  | **Statistic** | *-* | *z* = 1.24 | t(204) = 1.27 | t(333) = 0.62 |
|  | ***p*** | - | = 0.21 NS | = 0.21 NS | = 0.54 NS |
| **Control Group** | **Male** | 74 | - | 172.0 (158.0) | 88.3 (25.1) |
|  | **Female** | 100 | - | 196.3 (127.5) | 90.7 (20.9) |
|  | **Change (%)** | - | - | -14.1% | -2.7% |
|  | **Statistic** | *-* | *-* | t(172) = -1.12 | t(161) = -0.66 |
|  | ***p*** | - | - | = 0.26 NS | = 0.51 NS |
