# Supplementary material for: What would happen if twitter sent consequential messages to only a strategically important subset of users? A quantification of the Targeted Messaging Effect (TME)
Source: PLoS One. 2023 Jul 27;18(7):e0284495. doi: 10.1371/journal.pone.0284495 (PMC10374154; doi:10.1371/journal.pone.0284495)
Supplement: S4 Table — (DOCX) [file pone.0284495.s014.docx]

**S4 Table. Experiment 1: Demographic analysis by age.**

| **Condition** |  | ***n*** | **VMP (%)** | **Mean Search Time (sec) (SD)** | **Mean Scroll-Max Percentage (SD)** |
| --- | --- | --- | --- | --- | --- |
| **Bias Groups** | **≥ 33** | 231 | 83.8% | 202.4 (164.4) | 85.2 (23.8) |
|  | **< 33** | 127 | 77.4% | 167.7 (116.7) | 85.4 (25.7) |
|  | **Change (%)** | - | +7.6% | +17.1% | -0.2% |
|  | **Statistic** | *-* | *z* = 1.5 | t(356) = 2.11 | t(334) = -0.08 |
|  | ***p*** | - | = 0.14 NS | = 0.04 NS | = 0.93 NS |
| **Control Group** | **≥ 33** | 104 | - | 220.5 (161.3) | 88.3 (23.5) |
|  | **< 33** | 71 | - | 133.9 (82.1) | 91.7 (21.7) |
|  | **Change (%)** | - | - | +39.3% | -3.9% |
|  | **Statistic** | *-* | *-* | t(162) = 4.66 | t(162) = -0.96 |
|  | ***p*** | - | - | < .001 | = 0.34 NS |
