# Supplementary material for: What would happen if twitter sent consequential messages to only a strategically important subset of users? A quantification of the Targeted Messaging Effect (TME)
Source: PLoS One. 2023 Jul 27;18(7):e0284495. doi: 10.1371/journal.pone.0284495 (PMC10374154; doi:10.1371/journal.pone.0284495)
Supplement: S5 Table — (DOCX) [file pone.0284495.s015.docx]

**S5 Table. Experiment 1: Demographic analysis by race/ethnicity.**

| **Condition** |  | ***n*** | **VMP (%)** | **Mean Search Time (sec) (SD)** | **Mean Scroll-Max Percentage (SD)** |
| --- | --- | --- | --- | --- | --- |
| **Bias Groups** | **White** | 275 | 84.9% | 199.5 (149.8) | 85.2 (24.4) |
|  | **Non-White** | 83 | 70.7% | 158.9 (147.2) | 85.2 (25.0) |
|  | **Change (%)** | - | +16.7% | +20.4% | 0.0% |
|  | **Statistic** | *-* | *z* = 2.9 | t(356) = 2.18 | t(334) = -0.00 |
|  | ***p*** | - | < 0.01 | < 0.05 | = 1.0 NS |
| **Control Group** | **White** | 138 | - | 180.6 (142.5) | 89.3 (23.0) |
|  | **Non-White** | 37 | - | 203.4 (136.4) | 91.4 (22.0) |
|  | **Change (%)** | - | - | -12.6% | -2.4% |
|  | **Statistic** | *-* | *-* | t(173) = -0.87 | t(162) = -0.47 |
|  | ***p*** | - | - | = 0.38 NS | = 0.64 NS |
