# Supplementary material for: What would happen if twitter sent consequential messages to only a strategically important subset of users? A quantification of the Targeted Messaging Effect (TME)
Source: PLoS One. 2023 Jul 27;18(7):e0284495. doi: 10.1371/journal.pone.0284495 (PMC10374154; doi:10.1371/journal.pone.0284495)
Supplement: S6 Table — (DOCX) [file pone.0284495.s016.docx]

**S6 Table. Experiment 2: Demographic analysis by educational attainment.**

| **Condition** |  | ***n*** | **VMP (%)** | **Mean Search Time (sec) (SD)** | **Mean Scroll-Max Percentage (SD)** |
| --- | --- | --- | --- | --- | --- |
| **Bias Groups** | **≥ Bachelors** | 225 | 55.9% | 181.2 (111.6) | 87.7 (23.2) |
|  | **< Bachelors** | 157 | 65.4% | 194.4 (177.7) | 85.1 (24.4) |
|  | **Change (%)** | - | -17.0% | -7.3% | +3.0% |
|  | **Statistic** | *-* | *z* = -1.9 | t(241) = -0.82 | t(367) = 1.03 |
|  | ***p*** | - | = 0.06 NS | = 0.41 NS | = 0.30 NS |
| **Control Group** | **≥ Bachelors** | 85 | ­­­- | 209.4 (207.0) | 92.5 (17.9) |
|  | **< Bachelors** | 63 | - | 192.2 (158.0) | 85.6 (26.1) |
|  | **Change (%)** | - | - | +8.2% | +7.5% |
|  | **Statistic** | *-* | *-* | t(146) = 0.55 | t(95) = 1.72 |
|  | ***p*** | - | - | = 0.58 NS | = 0.09 NS |
