# Supplementary material for: What would happen if twitter sent consequential messages to only a strategically important subset of users? A quantification of the Targeted Messaging Effect (TME)
Source: PLoS One. 2023 Jul 27;18(7):e0284495. doi: 10.1371/journal.pone.0284495 (PMC10374154; doi:10.1371/journal.pone.0284495)
Supplement: S7 Table — (DOCX) [file pone.0284495.s017.docx]

**S7 Table. Experiment 2: Demographic analysis by gender.**

| **Condition** |  | ***n*** | **VMP (%)** | **Mean Search Time (sec) (SD)** | **Mean Scroll-Max Percentage (SD)** |
| --- | --- | --- | --- | --- | --- |
| **Bias Groups** | **Male** | 142 | 59.2% | 153.1 (99.6) | 86.6 (24.6) |
|  | **Female** | 235 | 62.2% | 204.9 (160.5) | 86.7 (23.1) |
|  | **Change (%)** | - | -5.1% | -33.8% | -0.1% |
|  | **Statistic** | *-* | *z* = -0.58 | t(375) = -3.87 | t(362) = -0.01 |
|  | ***p*** | - | = 0.56 NS | < .001 | = 1.0 NS |
| **Control Group** | **Male** | 52 | - | 177.4 (179.1) | 89.2 (22.1) |
|  | **Female** | 96 | - | 211.8 (189.8) | 89.8 (21.9) |
|  | **Change (%)** | - | - | -19.4% | -0.7% |
|  | **Statistic** | *-* | *-* | t(146) = -1.08 | t(135) = -0.15 |
|  | ***p*** | - | - | = 0.28 NS | = 0.88 NS |
