# Supplementary material for: What would happen if twitter sent consequential messages to only a strategically important subset of users? A quantification of the Targeted Messaging Effect (TME)
Source: PLoS One. 2023 Jul 27;18(7):e0284495. doi: 10.1371/journal.pone.0284495 (PMC10374154; doi:10.1371/journal.pone.0284495)
Supplement: S8 Table — (DOCX) [file pone.0284495.s018.docx]

**S8 Table. Experiment 2: Demographic analysis by age.**

| **Condition** |  | ***n*** | **VMP (%)** | **Mean Search Time (sec) (SD)** | **Mean Scroll-Max Percentage (SD)** |
| --- | --- | --- | --- | --- | --- |
| **Bias Groups** | **≥ 33** | 227 | 61.1% | 203.4 (147.8) | 89.1 (21.6) |
|  | **< 33** | 156 | 58.5% | 161.4 (130.6) | 83.2 (25.9) |
|  | **Change (%)** | - | +4.26% | +20.8% | +6.6% |
|  | **Statistic** | - | *z* = 0.51 | t(381) = 2.87 | t(287) = 2.32 |
|  | ***p*** | - | = 0.61 NS | < 0.01 | < 0.05 |
| **Control Group** | **≥ 33** | 100 | - | 219.3 (207.1) | 88.8 (22.7) |
|  | **< 33** | 49 | - | 165.0 (130.9) | 91.3 (20.3) |
|  | **Change (%)** | - | - | +24.8% | -2.8% |
|  | **Statistic** | *-* | *-* | t(147) = 1.68 | t(136) = -0.63 |
|  | ***p*** | - | - | = 0.10 NS | = 0.53 NS |
