# Supplementary material for: What would happen if twitter sent consequential messages to only a strategically important subset of users? A quantification of the Targeted Messaging Effect (TME)
Source: PLoS One. 2023 Jul 27;18(7):e0284495. doi: 10.1371/journal.pone.0284495 (PMC10374154; doi:10.1371/journal.pone.0284495)
Supplement: S9 Table — (DOCX) [file pone.0284495.s019.docx]

**S9 Table. Experiment 2: Demographic analysis by race/ethnicity.**

| **Condition** |  | ***n*** | **VMP (%)** | **Mean Search Time (sec) (SD)** | **Mean Scroll-Max Percentage (SD)** |
| --- | --- | --- | --- | --- | --- |
| **Bias Groups** | **White** | 296 | 58.0% | 191.3 (147.2) | 86.8 (23.7) |
|  | **Non-White** | 87 | 67.3% | 169.2 (123.7) | 86.4 (23.6) |
|  | **Change (%)** | - | -16.0% | +11.6% | +0.5% |
|  | **Statistic** | *-* | *z* = -1.56 | t(381) = 1.28 | t(368) = 0.11 |
|  | ***p*** | - | = 0.12 NS | = 0.20 NS | = 0.91 NS |
| **Control Group** | **White** | 114 | - | 206.1 (199.7) | 88.1 (23.1) |
|  | **Non-White** | 35 | - | 186.5 (138.2) | 94.2 (16.8) |
|  | **Change (%)** | - | - | +9.5% | -6.5% |
|  | **Statistic** | *-* | *-* | t(147) = 0.54 | t(77) = -1.66 |
|  | ***p*** | - | - | = 0.59 NS | = 0.10 NS |
