# Supplementary material for: What would happen if twitter sent consequential messages to only a strategically important subset of users? A quantification of the Targeted Messaging Effect (TME)
Source: PLoS One. 2023 Jul 27;18(7):e0284495. doi: 10.1371/journal.pone.0284495 (PMC10374154; doi:10.1371/journal.pone.0284495)
Supplement: S10 Table — (DOCX) [file pone.0284495.s020.docx]

**S10 Table. Experiment 3: Demographic analysis by educational attainment.**

| **Condition** |  | ***n*** | **VMP (%)** | **Mean Search Time (sec) (SD)** | **Mean Scroll-Max Percentage (SD)** |
| --- | --- | --- | --- | --- | --- |
| **Bias Groups** | **≥ Bachelors** | 213 | 79.1% | 160.1 (106.6) | 88.2 (22.7) |
|  | **< Bachelors** | 143 | 59.8% | 178.5 (124.0) | 85.6 (24.5) |
|  | **Change (%)** | - | +24.4% | -11.5% | +2.9% |
|  | **Statistic** | *-* | *z* = 3.95 | t(273) = -1.45 | t(328) = 1.00 |
|  | ***p*** | - | < 0.001 | = 0.15 NS | = 0.32 NS |
| **Control Group** | **≥ Bachelors** | 112 | ­­­- | 163.2 (75.4) | 92.5 (19.0) |
|  | **< Bachelors** | 70 | - | 167.8 (104.6) | 90.7 (19.1) |
|  | **Change (%)** | - | - | -2.8% | +1.9% |
|  | **Statistic** | *-* | *-* | t(114) = -0.32 | t(168) = 0.59 |
|  | ***p*** | - | - | = 0.75 NS | = 0.56 NS |
