# Supplementary material for: What would happen if twitter sent consequential messages to only a strategically important subset of users? A quantification of the Targeted Messaging Effect (TME)
Source: PLoS One. 2023 Jul 27;18(7):e0284495. doi: 10.1371/journal.pone.0284495 (PMC10374154; doi:10.1371/journal.pone.0284495)
Supplement: S11 Table — (DOCX) [file pone.0284495.s021.docx]

**S11 Table. Experiment 3: Demographic analysis by gender.**

| **Condition** |  | ***n*** | **VMP (%)** | **Mean Search Time (sec) (SD)** | **Mean Scroll-Max Percentage (SD)** |
| --- | --- | --- | --- | --- | --- |
| **Bias Groups** | **Male** | 133 | 66.7% | 147.8 (100.7) | 87.9 (22.9) |
|  | **Female** | 218 | 72.0% | 179.2 (120.2) | 86.4 (24.0) |
|  | **Change (%)** | - | -7.9% | -21.2% | +1.7% |
|  | **Statistic** | *-* | *z* = -1.05 | t(316) = -2.63 | t(323) = 0.56 |
|  | ***p*** | - | = 0.29 NS | < 0.01 | = 0.58 NS |
| **Control Group** | **Male** | 79 | - | 145.2 (81.4) | 90.3 (21.9) |
|  | **Female** | 100 | - | 180.1 (90.8) | 92.7 (17.0) |
|  | **Change (%)** | - | - | -24.0% | -2.7% |
|  | **Statistic** | *-* | *-* | t(177) = -2.67 | t(165) = -0.82 |
|  | ***p*** | - | - | < 0.01 | = 0.41 NS |
