# Supplementary material for: What would happen if twitter sent consequential messages to only a strategically important subset of users? A quantification of the Targeted Messaging Effect (TME)
Source: PLoS One. 2023 Jul 27;18(7):e0284495. doi: 10.1371/journal.pone.0284495 (PMC10374154; doi:10.1371/journal.pone.0284495)
Supplement: S12 Table — (DOCX) [file pone.0284495.s022.docx]

**S12 Table. Experiment 3: Demographic analysis by age.**

| **Condition** |  | ***n*** | **VMP (%)** | **Mean Search Time (sec) (SD)** | **Mean Scroll-Max Percentage (SD)** |
| --- | --- | --- | --- | --- | --- |
| **Bias Groups** | **≥ 33** | 221 | 71.2% | 185.4 (119.1) | 88.0 (22.8) |
|  | **< 33** | 136 | 69.3% | 138.4 (98.7) | 85.9 (24.4) |
|  | **Change (%)** | - | +2.7% | +25.4% | +2.39% |
|  | **Statistic** | *-* | *z* = 0.04 | t(325) = 4.03 | t(329) = 0.80 |
|  | ***p*** | - | = 0.70 NS | < 0.001 | = 0.43 NS |
| **Control Group** | **≥ 33** | 116 | - | 176.8 (84.2) | 92.5 (18.1) |
|  | **< 33** | 66 | - | 144.2 (90.2) | 90.7 (20.5) |
|  | **Change (%)** | - | - | +18.4% | +1.9% |
|  | **Statistic** | *-* | *-* | t(180) = 2.45 | t(168) = 0.61 |
|  | ***p*** | - | - | < 0.05 | = 0.55 |
