# Supplementary material for: What would happen if twitter sent consequential messages to only a strategically important subset of users? A quantification of the Targeted Messaging Effect (TME)
Source: PLoS One. 2023 Jul 27;18(7):e0284495. doi: 10.1371/journal.pone.0284495 (PMC10374154; doi:10.1371/journal.pone.0284495)
Supplement: S13 Table — (DOCX) [file pone.0284495.s023.docx]

**S13 Table. Experiment 3: Demographic analysis by race/ethnicity.**

| **Condition** |  | ***n*** | **VMP (%)** | **Mean Search Time (sec) (SD)** | **Mean Scroll-Max Percentage (SD)** |
| --- | --- | --- | --- | --- | --- |
| **Bias Groups** | **White** | 279 | 65.2% | 170.2 (116.2) | 86.7 (23.7) |
|  | **Non-White** | 78 | 92.1% | 157.8 (105.4) | 88.7 (22.5) |
|  | **Change (%)** | - | -41.3% | +7.9% | -2.3% |
|  | **Statistic** | *-* | *z* = -4.63 | t(134) = 0.90 | t(329) = -0.64 |
|  | ***p*** | - | < 0.001 | = 0.37 NS | = 0.53 NS |
|  |  |  |  |  |  |
| **Control Group** | **White** | 148 | - | 168.5 (89.4) | 91.3 (19.5) |
|  | **Non-White** | 34 | - | 149.8 (78.5) | 94.4 (16.5) |
|  | **Change (%)** | - | - | +11.1% | -3.4% |
|  | **Statistic** | *-* | *-* | t(180) = 1.12 | t(168) = -0.82 |
|  | ***p*** | - | - | = 0.26 NS | = 0.41 NS |
