# Supplementary material for: What would happen if twitter sent consequential messages to only a strategically important subset of users? A quantification of the Targeted Messaging Effect (TME)
Source: PLoS One. 2023 Jul 27;18(7):e0284495. doi: 10.1371/journal.pone.0284495 (PMC10374154; doi:10.1371/journal.pone.0284495)
Supplement: S14 Table — (DOCX) [file pone.0284495.s024.docx]

**S14 Table. Experiment 4: Demographic analysis by educational attainment.**

| **Condition** |  | ***n*** | **VMP (%)** | | **Mean Search Time (sec) (SD)** | **Mean Scroll-Max Percentage (SD)** |
| --- | --- | --- | --- | --- | --- | --- |
| **Bias Groups** | **≥ Bachelors** | 254 | | 37.1% | 174.4 (115.9) | 90.8 (21.2) |
|  | **< Bachelors** | 171 | | 32.6% | 182.6 (128.3) | 90.8 (20.5) |
|  | **Change (%)** | - | | +12.1% | -4.7% | 0.0% |
|  | **Statistic** | *-* | | *z* = 0.95 | t(423) = -0.69 | t(397) = 0.04 |
|  | ***p*** | - | | = 0.34 NS | = 0.49 NS | = 0.97 NS |
| **Control Group** | **≥ Bachelors** | 64 | | ­­­- | 171.6 (113.4) | 93.2 (16.6) |
|  | **< Bachelors** | 40 | | - | 181.6 (110.9) | 87.7 (27.4) |
|  | **Change (%)** | - | | - | -5.8% | +5.9% |
|  | **Statistic** | *-* | | *-* | t(102) = -0.44 | t(51) = 1.08 |
|  | ***p*** | - | | - | = 0.66 NS | = 0.29 NS |
