# Supplementary material for: What would happen if twitter sent consequential messages to only a strategically important subset of users? A quantification of the Targeted Messaging Effect (TME)
Source: PLoS One. 2023 Jul 27;18(7):e0284495. doi: 10.1371/journal.pone.0284495 (PMC10374154; doi:10.1371/journal.pone.0284495)
Supplement: S15 Table — (DOCX) [file pone.0284495.s025.docx]

**S15 Table. Experiment 4: Demographic analysis by gender.**

| **Condition** |  | ***n*** | **VMP (%)** | **Mean Search Time (sec) (SD)** | **Mean Scroll-Max Percentage (SD)** |
| --- | --- | --- | --- | --- | --- |
| **Bias Groups** | **Male** | 162 | 26.5% | 170.6 (138.0) | 90.6 (21.6) |
|  | **Female** | 259 | 38.9% | 184.0 (109.0) | 91.6 (19.8) |
|  | **Change (%)** | - | -46.8% | -7.9% | -1.1% |
|  | **Statistic** | *-* | *z* = -2.61 | t(419) = -1.10 | t(393) = -0.45 |
|  | ***p*** | - | < 0.01 | = 0.27 NS | = 0.65 NS |
| **Control Group** | **Male** | 43 | - | 160.3 (108.5) | 88.0 (25.3) |
|  | **Female** | 60 | - | 185.5 (115.0) | 93.2 (18.0) |
|  | **Change (%)** | - | - | -15.7% | -5.9% |
|  | **Statistic** | *-* | *-* | t(101) = -1.12 | t(66) = -1.10 |
|  | ***p*** | - | - | = 0.27 NS | = 0.28 NS |
