# Supplementary material for: What would happen if twitter sent consequential messages to only a strategically important subset of users? A quantification of the Targeted Messaging Effect (TME)
Source: PLoS One. 2023 Jul 27;18(7):e0284495. doi: 10.1371/journal.pone.0284495 (PMC10374154; doi:10.1371/journal.pone.0284495)
Supplement: S16 Table — (DOCX) [file pone.0284495.s026.docx]

**S16 Table. Experiment 4: Demographic analysis by age.**

| **Condition** |  | ***n*** | **VMP (%)** | **Mean Search Time (sec) (SD)** | **Mean Scroll-Max Percentage (SD)** |
| --- | --- | --- | --- | --- | --- |
| **Bias Groups** | **≥ 33** | 251 | 40.7% | 198.0 (134.6) | 93.2 (18.2) |
|  | **< 33** | 174 | 28.3% | 148.5 (90.8) | 87.6 (23.9) |
|  | **Change (%)** | - | +30.5% | +33.3% | +6.4% |
|  | **Statistic** | *-* | *z* = 2.62 | t(423) = 4.53 | t(299) = 2.55 |
|  | ***p*** | - | < 0.01 | < 0.001 | < 0.05 |
| **Control Group** | **≥ 33** | 61 | - | 187.4 (115.3) | 92.9 (18.5) |
|  | **< 33** | 43 | - | 158.6 (106.2) | 88.4 (25.1) |
|  | **Change (%)** | - | - | +15.4% | +4.8% |
|  | **Statistic** | *-* | *-* | t(102) = 1.29 | t(63) = 0.94 |
|  | ***p*** | - | - | = 0.20 NS | = 0.35 NS |
