# Supplementary material for: What would happen if twitter sent consequential messages to only a strategically important subset of users? A quantification of the Targeted Messaging Effect (TME)
Source: PLoS One. 2023 Jul 27;18(7):e0284495. doi: 10.1371/journal.pone.0284495 (PMC10374154; doi:10.1371/journal.pone.0284495)
Supplement: S17 Table — (DOCX) [file pone.0284495.s027.docx]

**S17 Table. Experiment 4: Demographic analysis by race/ethnicity.**

| **Condition** |  | ***n*** | **VMP (%)** | **Mean Search Time (sec) (SD)** | **Mean No. of Results Clicked (SD)** |
| --- | --- | --- | --- | --- | --- |
| **Bias Groups** | **White** | 337 | 36.3% | 174.7 (122.7) | 113.3 (76.0) |
|  | **Non-White** | 88 | 32.0% | 159.2 (99.5) | 111.2 (68.4) |
|  | **Change (%)** | - | +11.8% | +8.9% | +1.9% |
|  | **Statistic** | *-* | *z* = 0.75 | t(423) = 0.95 | t(114) = 1.55 |
|  | ***p*** | - | = 0.45 NS | = 0.34 NS | = 0.12 NS |
| **Control Group** | **White** | 79 | - | 173.4 (110.1) | 107.0 (52.2) |
|  | **Non-White** | 25 | - | 187.3 (118.1) | 107.6 (87.7) |
|  | **Change (%)** | - | - | -8.1% | -0.6% |
|  | **Statistic** | *-* | *-* | t(102) = -0.60 | t(24) = 0.81 |
|  | ***p*** | - | - | = 0.55 NS | = 0.43 NS |
