# Supplementary material for: What would happen if twitter sent consequential messages to only a strategically important subset of users? A quantification of the Targeted Messaging Effect (TME)
Source: PLoS One. 2023 Jul 27;18(7):e0284495. doi: 10.1371/journal.pone.0284495 (PMC10374154; doi:10.1371/journal.pone.0284495)
Supplement: S18 Table — (DOCX) [file pone.0284495.s028.docx]

**S18 Table. Experiment 1: Pre-and post-manipulation opinions by group.**

| **Pre-manipulation** |  | **Group 1**  **Pro-Morrison**  **Mean (*SD*)** | **Group 2**  **Pro-Shorten**  **Mean (*SD)*** | **Group 3**  **Control**  **Mean (*SD*)** | **Kruskal-**  **Wallis *H*** | ***p*** |
| --- | --- | --- | --- | --- | --- | --- |
|  | Impression of Morrison | 6.89 (1.91) | 7.02 (1.80) | 7.25 (1.77) | 3.30 | 0.19 NS |
|  | Likeability of Morrison | 7.05 (1.78) | 7.11 (1.88) | 7.36 (1.73) | 2.95 | 0.23 NS |
|  | Trust of Morrison | 6.01 (2.01) | 6.01 (1.96) | 6.35 (1.96) | 4.53 | 0.10 NS |
|  | Impression of Shorten | 7.11 (1.87) | 7.25 (1.71) | 7.15 (1.89) | 0.28 | 0.87 NS |
|  | Likeability of Shorten | 6.78 (1.77) | 7.14 (1.83) | 6.95 (1.77) | 3.67 | 0.16 NS |
|  | Trust of Shorten | 6.02 (2.07) | 6.18 (2.11) | 6.16 (2.06) | 0.65 | 0.72 NS |
| **Post-manipulation** |  |  |  |  |  |  |
|  | Impression of Morrison | 7.54 (1.98) | 3.96 (2.03) | 7.34 (1.85) | 215.05 | < 0.001 |
|  | Likeability of Morrison | 7.37 (1.97) | 4.14 (2.22) | 7.23 (1.82) | 183.38 | < 0.001 |
|  | Trust of Morrison | 6.90 (2.17) | 3.63 (2.14) | 6.75 (1.98) | 178.79 | < 0.001 |
|  | Impression of Shorten | 3.94 (2.07) | 7.72 (2.07) | 7.20 (1.78) | 212.10 | < 0.001 |
|  | Likeability of Shorten | 4.18 (2.16) | 7.42 (2.11) | 7.04 (1.76) | 174.68 | < 0.001 |
|  | Trust of Shorten | 3.77 (2.16) | 6.96 (2.22) | 6.53 (2.02) | 158.75 | < 0.001 |
