# Supplementary material for: What would happen if twitter sent consequential messages to only a strategically important subset of users? A quantification of the Targeted Messaging Effect (TME)
Source: PLoS One. 2023 Jul 27;18(7):e0284495. doi: 10.1371/journal.pone.0284495 (PMC10374154; doi:10.1371/journal.pone.0284495)
Supplement: S19 Table — (DOCX) [file pone.0284495.s029.docx]

**S19 Table. Experiment 2: Pre-and post-manipulation opinions by group.**

| **Pre-manipulation** |  | **Group 1**  **Pro-Morrison**  **Mean (*SD*)** | **Group 2**  **Pro-Shorten**  **Mean (*SD*)** | **Group 3**  **Control**  **Mean (*SD*)** | **Kruskal-**  **Wallis *H*** | ***p*** |
| --- | --- | --- | --- | --- | --- | --- |
|  | Impression of Morrison | 7.01 (1.64) | 7.02 (1.79) | 6.80 (1.86) | 1.51 | 0.47 NS |
|  | Likeability of Morrison | 7.04 (1.67) | 7.05 (1.80) | 6.90 (1.93) | 0.43 | 0.81 NS |
|  | Trust of Morrison | 6.03 (1.97) | 6.09 (2.03) | 5.89 (1.87) | 1.13 | 0.57 NS |
|  | Impression of Shorten | 7.08 (1.72) | 7.18 (1.85) | 6.91 (1.91) | 1.64 | 0.44 NS |
|  | Likeability of Shorten | 6.86 (1.58) | 6.93 (1.88) | 6.62 (1.84) | 2.58 | 0.28 NS |
|  | Trust of Shorten | 6.00 (1.97) | 6.18 (2.05) | 5.89 (1.91) | 2.65 | 0.27 NS |
| **Post-manipulation** |  |  |  |  |  |  |
|  | Impression of Morrison | 7.65 (1.75) | 3.89 (2.02) | 6.63 (1.83) | 226.54 | < 0.001 |
|  | Likeability of Morrison | 7.46 (1.71) | 3.98 (2.13) | 6.60 (1.80) | 202.04 | < 0.001 |
|  | Trust of Morrison | 7.05 (2.02) | 3.55 (2.00) | 6.14 (1.93) | 196.10 | < 0.001 |
|  | Impression of Shorten | 4.02 (2.03) | 7.52 (2.01) | 6.89 (1.66) | 214.19 | < 0.001 |
|  | Likeability of Shorten | 4.17 (2.05) | 7.26 (1.92) | 6.67 (1.78) | 184.85 | < 0.001 |
|  | Trust of Shorten | 3.72 (2.08) | 6.76 (2.12) | 6.21 (2.01) | 165.48 | < 0.001 |
