# Supplementary material for: What would happen if twitter sent consequential messages to only a strategically important subset of users? A quantification of the Targeted Messaging Effect (TME)
Source: PLoS One. 2023 Jul 27;18(7):e0284495. doi: 10.1371/journal.pone.0284495 (PMC10374154; doi:10.1371/journal.pone.0284495)
Supplement: S20 Table — (DOCX) [file pone.0284495.s030.docx]

**S20 Table. Experiment 3: Pre-and post-manipulation opinions by group.**

| **Pre-manipulation** |  | **Group 1**  **Pro-Morrison**  **Mean (*SD*)** | **Group 2**  **Pro-Shorten**  **Mean (*SD*)** | **Group 3**  **Control**  **Mean (*SD*)** | **Kruskal-**  **Wallis *H*** | ***p*** |
| --- | --- | --- | --- | --- | --- | --- |
|  | Impression of Morrison | 6.85 (1.60) | 6.94 (1.66) | 7.01 (1.78) | 0.92 | 0.63 NS |
|  | Likeability of Morrison | 6.93 (1.74) | 6.96 (1.81) | 7.03 (1.82) | 0.43 | 0.81 NS |
|  | Trust of Morrison | 6.22 (1.92) | 5.76 (1.87) | 6.07 (1.92) | 7.99 | 0.02 NS |
|  | Impression of Shorten | 6.94 (1.83) | 7.19 (1.70) | 7.23 (1.79) | 2.48 | 0.29 NS |
|  | Likeability of Shorten | 6.68 (1.91) | 6.88 (1.80) | 6.92 (1.94) | 1.45 | 0.48 NS |
|  | Trust of Shorten | 6.15 (2.12) | 5.82 (1.85) | 6.11 (2.03) | 4.02 | 0.13 NS |
| **Post-manipulation** |  |  |  |  |  |  |
|  | Impression of Morrison | 7.59 (1.92) | 3.50 (1.90) | 6.91 (2.02) | 238.18 | < 0.001 |
|  | Likeability of Morrison | 7.38 (1.96) | 3.42 (1.84) | 6.93 (1.97) | 236.73 | < 0.001 |
|  | Trust of Morrison | 6.88 (2.32) | 3.05 (1.88) | 6.22 (2.13) | 202.78 | < 0.001 |
|  | Impression of Shorten | 3.78 (2.02) | 7.86 (1.85) | 6.95 (1.92) | 235.87 | < 0.001 |
|  | Likeability of Shorten | 3.92 (2.08) | 7.56 (1.98) | 6.79 (2.04) | 195.46 | < 0.001 |
|  | Trust of Shorten | 3.33 (2.04) | 7.01 (2.14) | 6.30 (2.07) | 196.92 | < 0.001 |
