# Supplementary material for: What would happen if twitter sent consequential messages to only a strategically important subset of users? A quantification of the Targeted Messaging Effect (TME)
Source: PLoS One. 2023 Jul 27;18(7):e0284495. doi: 10.1371/journal.pone.0284495 (PMC10374154; doi:10.1371/journal.pone.0284495)
Supplement: S21 Table — (DOCX) [file pone.0284495.s031.docx]

**S21 Table. Experiment 4: Pre-and post-manipulation opinions by group.**

| **Pre-manipulation** |  | | **Group 1**  **Pro-Morrison; Positive Targeted Message;**  **Mean *SD*)** | | **Group 2**  **Pro-Shorten; Positive Targeted Message;**  **Mean *SD*)** | | **Group 3**  **Pro-Morrison; Negative Targeted Message;**  **Mean *SD*)** | | **Group 4**  **Pro-Shorten;**  **Negative**  **Targeted Message;**  **Mean *SD*)** | | **Group 5**  **Control;**  **Mean (*SD)*** | **Kruskal-**  **Wallis *H*** | | | ***p*** |
| --- | --- | --- | --- | --- | --- | --- | --- | --- | --- | --- | --- | --- | --- | --- | --- |
|  | Impression of Morrison | 7.25 (1.95) | | 6.99 (1.87) | | 6.91 (1.88) | | 7.07 (1.96) | | 7.07 (1.73) | | | 3.08 | 0.54 NS | |
|  | Likeability of Morrison | 7.28 (1.95) | | 7.05 (1.92) | | 6.78 (1.90) | | 7.13 (1.90) | | 7.13 (1.72) | | | 4.92 | 0.30 NS | |
|  | Trust of Morrison | 6.31 (2.10) | | 6.09 (1.95) | | 6.02 (1.83) | | 6.12 (1.96) | | 6.08 (1.88) | | | 2.14 | 0.71 NS | |
|  | Impression of Shorten | 7.36 (1.93) | | 7.37 (1.80) | | 7.06 (1.82) | | 6.95 (1.93) | | 7.13 (1.78) | | | 4.49 | 0.34 NS | |
|  | Likeability of Shorten | 7.04 (1.88) | | 7.07 (1.86) | | 6.88 (1.84) | | 6.64 (1.89) | | 6.75 (1.85) | | | 4.59 | 0.33 NS | |
|  | Trust of Shorten | 6.35 (2.19) | | 6.29 (1.93) | | 6.25 (1.82) | | 6.06 (1.83) | | 6.23 (2.08) | | | 1.38 | 0.85 NS | |
| **Post-manipulation** |  |  | |  | |  | |  | |  | | |  |  | |
|  | Impression of Morrison | 7.14 (2.01) | | 6.90 (1.78) | | 6.93 (1.87) | | 5.14 (2.33) | | 6.84 (1.79) | | | 50.65 | < 0.001 | |
|  | Likeability of Morrison | 7.05 (2.05) | | 6.92 (1.85) | | 6.76 (1.87) | | 5.41 (2.12) | | 6.77 (1.94) | | | 41.22 | < 0.001 | |
|  | Trust of Morrison | 6.40 (2.09) | | 6.28 (2.00) | | 6.43 (2.09) | | 4.93 (2.20) | | 6.20 (2.07) | | | 34.96 | < 0.001 | |
|  | Impression of Shorten | 7.29 (1.90) | | 7.46 (1.77) | | 5.38 (1.99) | | 7.11 (1.95) | | 6.89 (1.87) | | | 65.99 | < 0.001 | |
|  | Likeability of Shorten | 7.08 (1.86) | | 7.31 (1.85) | | 5.45 (2.17) | | 6.92 (1.84) | | 6.74 (1.90) | | | 48.18 | < 0.001 | |
|  | Trust of Shorten | 6.53 (2.08) | | 6.74 (2.10) | | 4.97 (2.24) | | 6.44 (1.89) | | 6.38 (2.09) | | | 41.46 | < 0.001 | |
