# Supplementary material for: What would happen if twitter sent consequential messages to only a strategically important subset of users? A quantification of the Targeted Messaging Effect (TME)
Source: PLoS One. 2023 Jul 27;18(7):e0284495. doi: 10.1371/journal.pone.0284495 (PMC10374154; doi:10.1371/journal.pone.0284495)
Supplement: S22 Table — (DOCX) [file pone.0284495.s032.docx]

**S22 Table. Experiments 1-3: Pre- and post-manipulation votes on 11-point scale (-5 to +5).**

|  | **Group 1**  **Pro-Morrison;**  **Mean (*SD*)** | **Group 2**  **Pro-Shorten;**  **Mean (*SD*)** | **Group 3**  **Control;**  **Mean (*SD*)** | **Kruskal-**  **Wallis *H*** | ***p*** |
| --- | --- | --- | --- | --- | --- |
| **Experiment 1** | | | | | |
| Pre-Manipulation Vote | 0.43 (2.61) | 0.06 (2.76) | -0.35 (2.71) | 7.01 | 0.03 NS |
| Post-Manipulation Vote | -2.41 (2.47) | 2.69 (2.45) | -0.53 (2.99) | 206.00 | < 0.001 |
| **Experiment 2** | | | | | |
| Pre-Manipulation Vote | 0.08 (2.59) | 0.52 (2.60) | -0.09 (2.24) | 6.65 | 0.04 NS |
| Post-Manipulation Vote | -2.48 (2.47) | 2.62 (2.41) | 0.23 (2.59) | 227.45 | < 0.001 |
| **Experiment 3** | | | | | |
| Pre-Manipulation Vote | 0.24 (2.74) | 0.75 (2.64) | 0.24 (2.76) | 3.87 | 0.15 NS |
| Post-Manipulation Vote | -2.64 (2.25) | 3.19 (2.02) | 0.07 (2.93) | 259.78 | < 0.001 |
