# Supplementary material for: What would happen if twitter sent consequential messages to only a strategically important subset of users? A quantification of the Targeted Messaging Effect (TME)
Source: PLoS One. 2023 Jul 27;18(7):e0284495. doi: 10.1371/journal.pone.0284495 (PMC10374154; doi:10.1371/journal.pone.0284495)
Supplement: S23 Table — (DOCX) [file pone.0284495.s033.docx]

**S23 Table. Experiment 4: Pre- and post-manipulation vote on 11-point scale (-5 to +5).**

| **Experiment 4** |  |  |  |  |  |  |
| --- | --- | --- | --- | --- | --- | --- |
|  | **Group 1**  **Pro-Morrison; Positive Targeted Message;**  **Mean (*SD*)** | **Group 2**  **Pro-**  **Shorten; Positive Targeted Message;**  **Mean (*SD*)** | **Group 3**  **Pro-Morrison; Negative**  **Targeted Message;**  **Mean (*SD*)** | **Group 4**  **Pro-**  **Shorten; Negative**  **Targeted Message;**  **Mean (*SD*)** | **Kruskal-**  **Wallis *H*** | ***p*** |
| Pre-Manipulation Vote | 0.34 (2.63) | 0.85 (2.80) | 0.41 (2.70) | 0.11 (2.89) | 9.22 | 0.10 NS |
| Post-Manipulation Vote | -0.32 (2.87) | 1.25 (2.66) | -1.18 (2.80) | 1.46 (2.81) | 9.91 | < 0.01 |
