# Supplementary material for: What would happen if twitter sent consequential messages to only a strategically important subset of users? A quantification of the Targeted Messaging Effect (TME)
Source: PLoS One. 2023 Jul 27;18(7):e0284495. doi: 10.1371/journal.pone.0284495 (PMC10374154; doi:10.1371/journal.pone.0284495)
Supplement: S2 Text — (DOCX) [file pone.0284495.s035.docx]

**S2 Text. Experiments 1 to 4: Instructions immediately preceding Twitter simulation.**

Your task: Find out which candidate, if either, will do a better job of protecting Australia.

On the next page, we will show you a phone screen that will allow you to search through a Twitter feed showing comments people have made about protecting Australia from foreign threats. That feed will include information on how well the two candidates – Bill Shorten and Scott Morrison – might be able to protect Australia.

Your task is to use this Twitter feed to find out which candidate will do a better job of protecting Australia if he is elected Prime Minister. You might also conclude that they will do an equally good job.

Please scroll through the entire Twitter feed before making up your mind. Thanks!
