# Supplementary material for: What would happen if twitter sent consequential messages to only a strategically important subset of users? A quantification of the Targeted Messaging Effect (TME)
Source: PLoS One. 2023 Jul 27;18(7):e0284495. doi: 10.1371/journal.pone.0284495 (PMC10374154; doi:10.1371/journal.pone.0284495)
Supplement: S3 Text — (DOCX) [file pone.0284495.s036.docx]

**S3 Text. Experiments 1 to 4: Textual content and positions of the five targeted messages.**

*Position 2 (negative):* [Candidate name] caught spending taxpayer money on lush vacation in Mexico.

*Position 7 (positive):* [Candidate name] has been nominated for The Innovation in Politics Award, which recognizes creative politicians who have the courage to break new ground to find innovative solutions for today’s challenges.

*Position 12 (negative):* [Candidate name] charged with driving under the influence while vacationing in Adelaide.

*Position 25 (positive):* [Candidate name] awarded an honorary doctorate from the University of Melbourne, in recognition for his humanitarian efforts during the Australian wildfires.

*Position 31 (negative):* [Candidate name] under fire as tabloid photos reveal an extramarital affair with Australian model Christine Chen.
