# Supplementary material for: What would happen if twitter sent consequential messages to only a strategically important subset of users? A quantification of the Targeted Messaging Effect (TME)
Source: PLoS One. 2023 Jul 27;18(7):e0284495. doi: 10.1371/journal.pone.0284495 (PMC10374154; doi:10.1371/journal.pone.0284495)
Supplement: S4 Text — (DOCX) [file pone.0284495.s037.docx]

**S4 Text. Vote Manipulation Power (VMP) calculation.**

Vote Manipulation Power (VMP) is calculated as follows:

*p' – p*
 *p*

where *p* is the total number of people who voted for the favored candidate pre-manipulation, and *p'* is the total number of people who voted for the favored candidate post-manipulation. If, pre-manipulation, a group of 100 people is split 50/50 in the votes they give us, and if, post-manipulation, a total of 67 people now vote for the favored candidate, the VMP is

*67 – 50*
 *50*

or 34%. Because *p'* is 17 points larger than *p*, the win margin is 34 (2 x 17, or 34%), and the final vote is 67 to 33, with the favored candidate the winner. So in any group in which the vote is split 50/50 pre-manipulation, the VMP is also the win margin. Note that 17 individuals did not need to *shift* to produce this win margin. We only need the *net* number of people voting for the favored candidate to be 67.
